# Bladder cancer organoids as a functional system to model different disease stages and therapy response

**DOI:** 10.1038/s41467-023-37696-2

**Published:** 2023-04-18

**Authors:** Martina Minoli, Thomas Cantore, Daniel Hanhart, Mirjam Kiener, Tarcisio Fedrizzi, Federico La Manna, Sofia Karkampouna, Panagiotis Chouvardas, Vera Genitsch, Antonio Rodriguez-Calero, Eva Compérat, Irena Klima, Paola Gasperini, Bernhard Kiss, Roland Seiler, Francesca Demichelis, George N. Thalmann, Marianna Kruithof-de Julio

**Affiliations:** 1 Department for BioMedical Research, Urology Research Laboratory, University of Bern, 3008 Bern, Switzerland; 2 Department of Cellular, Computational and Integrative Biology, University of Trento, 38123 Trento, Italy; 3 Institute of Tissue Medicine and Pathology, University of Bern, 3008 Bern, Switzerland; 4 Department for BioMedical Research, University of Bern, 3008 Bern, Switzerland; 5 Department of Pathology, General Hospital, Medical University Vienna, 1090 Vienna, Austria; 6 Department of Urology, Inselspital, Bern University Hospital, 3010 Bern, Switzerland; 7 Department of Urology, Hospital Center Biel, 2501 Biel, Switzerland; 8 Department for BioMedical Research, Translation Organoid Research, University of Bern, 3008 Bern, Switzerland; 9 The Caryl and Israel Englander Institute for Precision Medicine, Weill Cornell Medicine, New York, NY 10021 USA; 10 Bern Center for Precision Medicine, Inselspital, Bern University Hospital, 3008 Bern, Switzerland

**Keywords:** Genetic databases, Bladder cancer, Cancer genomics

## Abstract

Bladder Cancer (BLCa) inter-patient heterogeneity is the primary cause of treatment failure, suggesting that patients could benefit from a more personalized treatment approach. Patient-derived organoids (PDOs) have been successfully used as a functional model for predicting drug response in different cancers. In our study, we establish PDO cultures from different BLCa stages and grades. PDOs preserve the histological and molecular heterogeneity of the parental tumors, including their multiclonal genetic landscapes, and consistently share key genetic alterations, mirroring tumor evolution in longitudinal sampling. Our drug screening pipeline is implemented using PDOs, testing standard-of-care and FDA-approved compounds for other tumors. Integrative analysis of drug response profiles with matched PDO genomic analysis is used to determine enrichment thresholds for candidate markers of therapy response and resistance. Finally, by assessing the clinical history of longitudinally sampled cases, we can determine whether the disease clonal evolution matched with drug response.

## Abstract (web summary)

Bladder cancer heterogeneity can limit treatment efficacy in individual patients. Here, the authors use patient derived organoids to develop a drug screening pipeline and identify markers of treatment response.

## Introduction

Bladder cancer (BLCa) is subdivided into two pathological classes, non-muscle invasive (NMIBC) and muscle-invasive (MIBC). Around 70% of the tumors are NMIBC and treated with transurethral resection of the bladder (TUR-B) and intravesical instillations of chemotherapy alone (i.e., mitomycin C or epirubicin) or in combination with Bacillus Calmette–Guérin (BCG) vaccine^1,2^. A significant fraction of tumors will recur, causing considerable morbidity and high costs^1^. Around 10% of NMIBC progress to MIBC following several recurrences^3^. 25–30% of patients are directly diagnosed with MIBC characterized by rapid local and systemic progression, resulting in high mortality^4,5^. MIBC patients are treated with systemic neoadjuvant cisplatin-based chemotherapy followed by radical cystectomy^6^. This treatment is highly invasive and beneficial for only 30–40% of patients^7^.

NMIBC and MIBC distinct clinical profiles can also be attributed to their genetic differences, confirmed by molecular classifications^8–11^. Molecular classifications have highlighted the substantial BLCa heterogeneity, which complicates the treatment, promoting tumor recurrence and progression. To improve BLCa outcomes, treatments should be tailored to each patient to identify more effective compounds. This requires the development of reliable customized models with properties close to the parental tumor (PT). In recent years, precision medicine approaches have also focused on patient-derived organoids (PDOs), which have been shown to recapitulate key aspects of tissue composition, including tumor architecture, heterogeneity, and function, and to remain genetically stable in culture^12,13^. PDOs proved to help predict drug response in vitro^14–20^.

In this work, we successfully establish and culture organoids from NMIBC and MIBC that recapitulate the PTs’ key aspects, such as histological and molecular heterogeneity and their multiclonal genetic landscapes. BLCa PDOs are implemented in a drug screening pipeline to test standard-of-care (SOC) drugs and FDA-approved compounds for other solid tumors. Integrative analysis of drug response profiles with matched PDO genomic analysis allows us to identify biomarkers/signatures potentially helpful in designing a treatment regimen unique to the patient’s genetic profile. Furthermore, by assessing longitudinal studies, the clonal evolution of the disease could be determined and matched with PDO drug responses. These results strengthen the evidence that BLCa PDOs can be applied as a platform for precision medicine.

## Results

### Establishment and culture of BLCa PDOs from diverse clinical samples

**Fig. 1:**
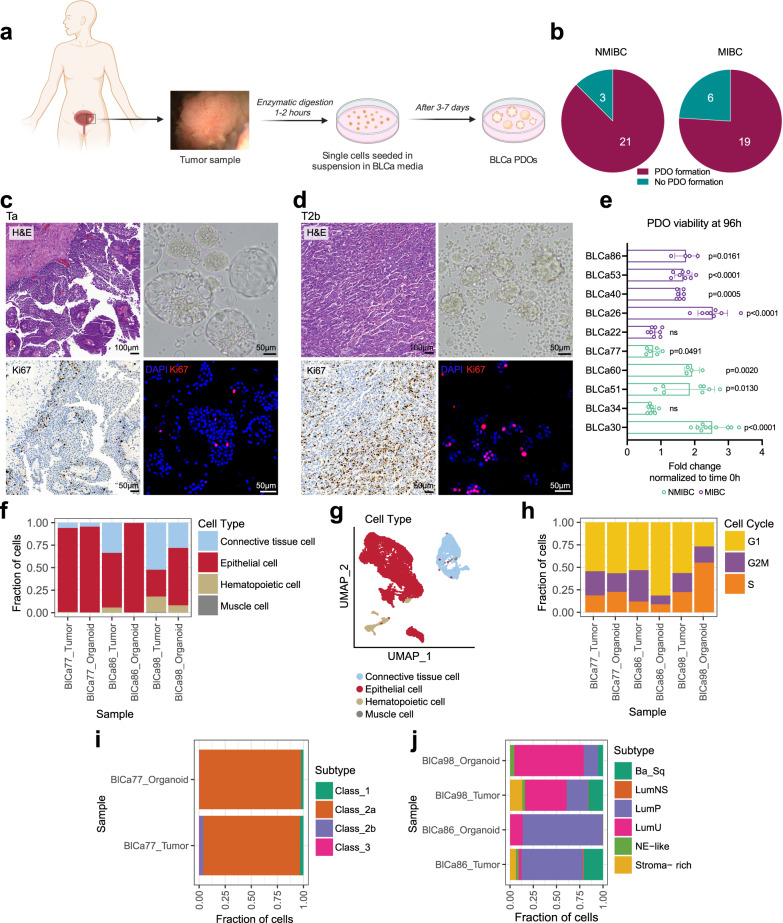
Isolation and culture of patient-derived organoids (PDOs) from non-muscle invasive bladder cancer (NMIBC) and muscle invasive bladder cancer (MIBC). **a** Scheme of the experimental protocol for bladder cancer (BLCa) organoids derivation and culture. Created with BioRender.com. **b** Number of PDO formation and no PDO formation over the total samples cultured for NMIBC (*n* = 24 biological samples) and MIBC (*n* = 25 biological samples). **c**, **d** Morphology of parental tumor (PTs, Hematoxylin and Eosin staining) and matched PDOs at passage (p) 1 (brightfield image, upper) for two representative cases (BLCa34, transurethral resection of bladder tumor, Ta stage (**c**); BLCa40, cystectomy, T2b stage (**d**)). Immunohistochemistry for Ki67 for PT, and whole-mount immunofluorescence staining for Ki67 for PDOs (bottom). **e** Viability assay of PDOs at p2 derived from 5 NMIBC and 5 MIBC samples after 96 h in culture. Each data point corresponds to one technical replicate (mean ± SD) at 96 h (normalized to the time 0 h) for one experiment (n represents the technical replicates: *n* = 10 for BLCa30 (96 h); *n* = 5 for BLCa22 (0 h), BLCa26 (0 h), BLCa30 (0 h), BLCa40 (0 h), BLCa51 (0 h), BLCa53 (0 h), and BLCa77 (0 h); *n* = 8 for BLCa34 (96 h), BLCa51 (96 h), BLCa22 (96 h), BLCa26 (96 h) and BLCa53 (96 h); *n* = 7 for BLCa40 (96 h), and BLCa86 (0 h); *n* = 6 for BLCa77 (96 h); *n* = 4 for BLCa60 (0 h and 96 h), BLCa86 (96 h), and BLCa34 (0 h)). Statistically significance between time 0 and 96 h was calculated by two-sided Welch’s test. **f** Fraction of cell types in PT/PDOs (p1) pairs for three representative cases (BLCa77 NMIBC, BLCa86 MIBC, and BLCa98 MIBC). **g** UMAP plot of cells derived from PT/PDOs (p1) pairs clustered by cell types. **h** Fraction of cells in cell cycle phases in PT/PDOs pairs. **i**, **j** Proportion of epithelial cells that correspond to each molecular class for the BLCa77 sample (**i**, UROMOL2021 classifier) and for BLCa86 and BLCa98 samples (**j**, Consensus classifier). Ba_Sq basal/squamous, LumNS luminal nonspecified, LumP luminal papillary, LumU luminal unstable, NE-like neuroendocrine-like, ns not significant.

PDOs were generated from specimens obtained from patients that underwent either TUR-B, cystectomy, or nephroureterectomy (Fig. 1a, Tables 1, 2, Supplementary Data 1) at the Inselspital, University Hospital in Bern and representing the spectrum of BLCa, ranging from low-grade (LG) non-invasive to high-grade (HG) invasive tumors, including both NMIBC and MIBC. PDOs were derived and grown in suspension^21^ from fresh and cryopreserved tissue and cryopreserved single cells.

**Table 1:** Pathological data of the tumor

Sample ID	Tumor stage	Pathological classification		Concomitant CIS	Immune infiltration	Main PDO morphology
		Papillary urothelial carcinoma	Urothelial carcinoma			
BLCa34	Ta	LG non-invasive	N/A	No	None	Hollow
BLCa35	Ta	HG non-invasive	N/A	No	Peritumoral weak	Solid
BLCa57	Ta	LG non-invasive	N/A	No	Peritumoral weak	Hollow
BLCa60	Ta	LG non-invasive	N/A	No	Peritumoral weak	Mixed
BLCa69	Ta	HG non-invasive	N/A	No	None	Mixed
BLCa81 (BLCa69)	Ta/Tis	HG non-invasive	N/A	Yes	None	Hollow
BLCa61	T1	HG non-invasive	HG invasive	No	Peritumoral weak	Hollow
BLCa82	T1	No	HG invasive with squamous differentiation (70% of tumor)	No	None	Solid
BLCa85	T1	HG non-invasive	HG invasive	No	None	Hollow
BLCa100	yT1	HG non-invasive	HG invasive	Yes	Intermediate	Mixed
BLCa112 (BLCa57)	T1	HG non-invasive	HG invasive	No	Intermediate	Mixed
BLCa46	T ≥ 1	HG non-invasive	HG invasive	No	Peritumoral weak	Solid
BLCa50	T ≥ 1	HG non-invasive with glandular differentiation (30% of the tumor)	HG invasive with glandular differentiation (30% of the tumor)	Yes	Intra- and peritumoral weak	Solid
BLCa22	T2a	HG non-invasive	HG invasive	No	Peritumoral weak	Solid
BLCa27	T2a	No	HG invasive	No	Weak	Solid
BLCa40 (BLCa35)	T2b	HG non-invasive	HG invasive with glandular differentiation (mucinous/singet ring cells, 20% tumor)	Yes	Intra- and peritumoral weak	Solid
BLCa86 (BLCa85)	T2b	HG non-invasive	HG invasive	No	Intermediate	Hollow
BLCa47	T3a	HG non-invasive	HG invasive with squamous differentiation (80% of tumor)	No	Intermediate	Solid
BLCa92	T3a	HG non-invasive	HG invasive	Yes	Intermediate	Solid
BLCa98	T3a	HG invasive	HG invasive	Yes	Intermediate	Solid
BLCa48	T3b	No	HG invasive with sarcomatoid/squamous differentiation (90% of tumor)	No	Intra- and peritumoral high	Solid
BLCa33	yT3b	HG non-invasive	HG invasive	No	Peritumoral weak	Mixed

Tumor stage and pathological classification with information regarding immune infiltration were evaluated for the parental tumor. If different samples are derived from the same patient at different time points, previous samples are reported in brackets in the Sample ID column. The main patient-derived organoid (PDO) morphology is reported in the last column.

*CIS or Tis* carcinoma in situ, *HG* high-grade, *LG* low-grade, *N/A* not available, *yT* post-therapy tumor stage.

**Table 2:** Summary of patients’ clinical history

Sample ID	Smoking status	Former recurrences (treatment) and comorbidities	Pre- operative treatment	Post-operative treatment	Treatment response	Progression and time to progression	Cancer-specific death
BLCa22	Active (30 PY)	BLCa (Ta), prostatic adenocarcinoma (T2c)	No	No	N/A	No	N/A
BLCa27	N/A	BLCa (Ta and T1, 6x Epirubicin instillation 6 x BCG instillation), prostate and lung cancer	No	No	N/A	210 days lymph nodes and lung mets	Yes
BLCa33	Former (2013)	Adenocarcinoma of the prostate (T1b)	4x NAC cisp/gem	No	Yes	No	N/A
BLCa34	Former (2009, 70-80 PY)	N/A	No	1x Epirubicin instillation	Yes	No	N/A
BLCa35	N/A	Incidental prostatic adenocarcinoma (T1a), hepatocellular carcinoma	No	No	N/A	No	No
BLCa40 (BLCa35)	N/A	BLCa35	No	No	N/A	No	No
BLCa46	No	N/A	No	6x BCG instillation	Yes	No	No
BLCa47	N/A	N/A	No	No	N/A	No	N/A
BLCa48	Former (until 1990, 30 PY)	Former BLCa (Ta, 6x BCG instillation, 3x tremelimumab and durvaalumab)	No	3x Adjuvant chemo cisp/gem	Yes	No	N/A
BLCa50	N/A	Prostate cancer (T2a)	No	No	N/A	No	N/A
BLCa57	N/A	Acinar prostatic adenocarcinoma (T2c), diagnosed with panurothelial disease (1x Epirubicin and 6x BCG instillation)	No	No	N/A	488 days (BLCa112)	N/A
BLCa112 (BLCa57)	N/A	BLCa57	No	No	N/A	No	N/A
BLCa60	No	Former BLCa (Ta and T1), prostatic adenocarcinoma (pT2a)	No	No	N/A	No	N/A
BLCa61	N/A	Former BLCa (Ta and T1, 1x BCG instillation)	No	No	No	463 days 1st relapse (uns., urethra), 793 days 2nd relapse (uns., urethra penis)	N/A
BLCa69	Former (2019, 40 PY)	Acute myeloid leukemia (BCR-ABL1)	No	1x Epirubicin instillation	No	46 days 1st relapse (uns., Ta HG, lateral left bladder wall), 197 days 2nd relapse (BLCa81)	N/A
BLCa81 (BLCa69)	BLCa69	BLCa69	No	6x BCG instillation	No	389 days from 1st (BLCa69) 3rd relapse (uns., Ta HG, posterior bladder wall), 446 days 4th relapse (sampled, Relapse 2, atypical urothelial, posterior bladder wall) followed by epirubicin treatment, 679 days 5th relapse (uns., Ta HG, penile urethra), 699 days 6th relapse (sampled, Relapse 3, Ta HG, urethra)	N/A
BLCa82	Active (70 PY)	N/A	No	Palliative chemo (carbo/gem)	No	Pulmonary and hepatic mets (post-operative)	N/A
BLCa85	N/A	Acinary prostatic adenocarcinoma (T1a)	No	No	N/A	No	N/A
BLCa86 (BLCa85)	N/A	BLCa85	No	No	N/A	No	N/A
BLCa92	N/A	N/A	No	No	No	314 days liver mets (palliative chemo carbo/gem)	N/A
BLCa98	No	Benign prostate hyperplasia	No	No	N/A	No	N/A
BLCa100	Active (60 PY)	Acinary prostatic adenocarcinoma (T1a), BLCa (Ta and Tis)	4x NAC cisp/gem	No	N/A	No	N/A

If different samples are derived from the same patient at different time points, previous samples are reported in brackets in the Sample ID column.

*BCG* Bacillus Calmette–Guérin treatment, *chemo* chemotherapy, *Cis/Gem* cisplatin and gemcitabine combination, *Carbo/Gem* carboplatin and gemcitabine combination, *Mets* metastases, *NAC* neaoadjuvant chemotherapy, *N/A* not available, *PY* packages per year, *uns.* unsampled, *1x* 1 cycle.

PDO cultures were successfully established from BLCa samples irrespective of tumor stage, grade or histological pattern, as determined by pathology reports (Fig. 1b, Supplementary Figs. 1, 2, Table 1, Supplementary Data 1). Although not significantly different, organoid-forming efficiency was moderately higher in NMIBC (21 out of 24, 87%) vs MIBC (19 out of 25, 76%, two-sided Fisher’s test, *p*-value = 0.4635, Fig. 1b). BLCa PDOs formed within 3 to 7 days, and failure to generate PDOs was mainly associated with an insufficient number of viable cells in the resection or contamination by microorganisms (see supplementary Data 1 for a detailed report).

The proliferative potential of PDO cultures was investigated and compared to the PT. PTs were grouped in low- and high-proliferation rate (≤ or >22% of Ki67^+^ nuclei per section) and correlated to a low (2% ± 2%) or high (12% ± 9%) percentage of Ki67^+^ cells in the corresponding PDOs (Fig. 1c, d, Supplementary Fig. 3a, b, Supplementary Table 1). In addition, organoids viability was investigated at 96 h post-seeding for a subset of 10 samples. 7 out of 10 samples showed a significant increase in viability at 96 h compared to the day of seeding. In contrast, the remaining samples did not increase their cell viability (Fig. 1e). No significant difference in viability was observed between NMIBC and MIBC organoids (unpaired two-sided Wilcoxon test, *p*-value >0.999, Fig. 1e).

Single-cell RNA sequencing was performed to compare PT and PDO cellular heterogeneity (*n* = 3, BLCa77 NMIBC, BLCa86 MIBC, BLCa98 MIBC, Fig. 1f–j). The fraction of epithelial cells was increased in PDOs compared to the PT, whereas the percentage of tumor microenvironment (TME) cells (i.e., connective tissue cells) was reduced (Fig. 1f). As expected, associated extracellular pathways were enriched in genes upregulated in the PT due to the depletion of TME cells in the PDOs (Supplementary Fig. 3c). Moreover, we showed that cells clustered based on cell types rather than on sample type (Fig. 1g, Supplementary Fig. 3d).

The proliferation potential of PDOs was further investigated with the scRNA data. Compared to the PT, PDOs presented a significantly higher fraction of cells in the S-phase of the cell cycle (Fig. 1h). In addition, they were positive to the proliferation markers Ki67 and PCNA (Supplementary Fig. 3e).

We further explored the cellular heterogeneity of PTs and PDOs pairs by comparing the fraction of epithelial cells positive for two subtype classifiers available for NMIBC (UROMOL2021 classifier) and MIBC (Consensus classifier, Fig. 1i, j). In all three samples, the PDOs showed an enrichment of the most predominant PT subtype. In particular, BLCa77 PDOs showed enrichment for Class 2a, BLCa86 PDOs for the LumP subtype, and BLCa98 PDOs for the LumU subtype (Fig. 1i, j). In addition, we compared the fraction of cells positive for different basal and luminal markers between matched samples pairs, and we observed that PDO sub-populations composition was consistent with one of the PTs (R > 0.6, Supplementary Fig. 3f). These results suggest the preservation of the primary molecular subtype/transcription between PT and PDOs.

### PDOs preserve key phenotypic and histological features of PTs

**Fig. 2:**
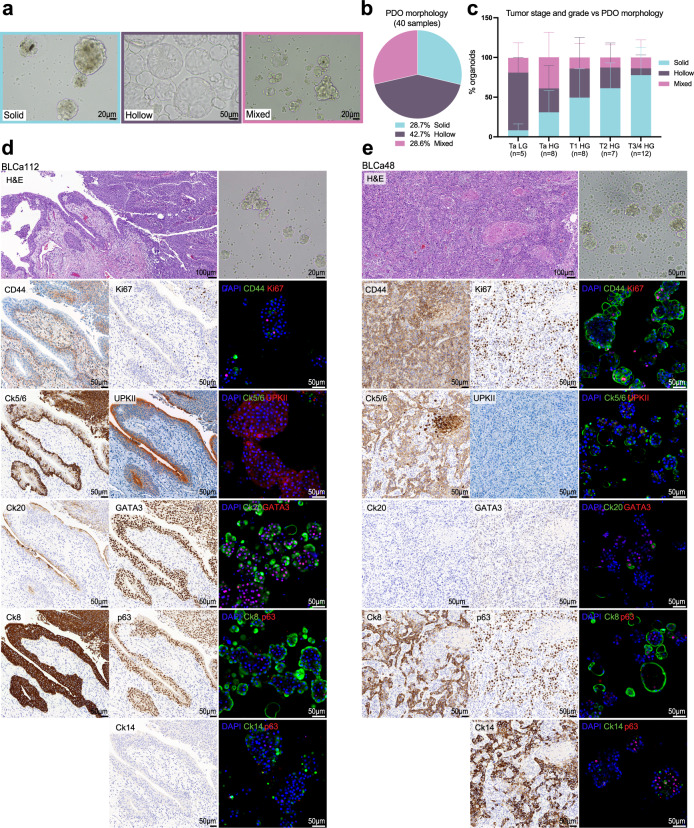
Bladder cancer (BLCa) patient-derived organoids (PDOs) recapitulate original primary tumor (PT) features in vitro. **a** Representative brightfield images of BLCa PDOs at passage (p) 1 with a solid (BLCa50, day 9), hollow (BLCa34, day 7), or mixed (BLCa69, day 7) morphology. **b** % of PDO morphology over the total analyzed samples (*n* = 1763 total counted organoids from 40 biological samples). **c** Distribution of PDO morphology in samples grouped based on PT stage and grade (mean from biological samples ± SD; *n* represents the number of biological samples: *n* = 5 for Ta LG; *n* = 8 for Ta HG and T1 HG; *n* = 7 for T2 HG; and *n* = 12 for T3/4 HG). Two-way ANOVA test with Tukey’s multiple comparison (matching values of each biological sample stacked into sub-columns) was used to compare the % of PDO morphologies between tumor stages and grades (Solid: Ta LG vs T3/4 *p*-value = 0.0001; Ta HG vs T3/4 *p*-value = 0.0256 Ta HG vs T2 *p*-value = 0.0237. Hollow: Ta HG vs T3/4 *p*-value = 0.0026). **d**, **e** Hematoxylin and Eosin staining and immunohistochemistry staining of PT for indicated markers and brightfield images and whole-mount immunofluorescent staining of PDOs at p1 for indicated markers. One representative sample for non-muscle invasive BLCa (BLCa112, **d**) and one for muscle invasive BLCa (BLCa48, **e**). Ck cytokeratin, HG high-grade, LG low-grade, UPKII uroplakin II.

PDOs were characterized by morphological analyses and marker expression (Table 1) and grouped into three morphological patterns: solid, hollow, or mixed (Fig. 2a). Solid morphology was defined by cellular aggregates lacking a luminal space. Hollow organoids presented a luminal space delimited either by a thin layer of cells or by an organized layer of epithelium. Mixed morphology included samples presenting a mix of solid and hollow features or with more heterogeneous morphologies, i.e., hybrid and budding organoids. Hybrid organoids exhibited both hollow and solid phenotypes. In contrast, budding organoids presented budding structures on the surface (Supplementary Fig. 4). 28.7% of the analyzed organoids were solid, 42.7% were hollow, and the remaining 28.6% were mixed (Fig. 2b).

We then investigated the correlation between PDO morphology and PT stage and grade (Fig. 2c). Organoids with a predominant hollow morphology significantly originated more frequently from Ta LG tumors (*n* = 4/5, 72% ± 19%) compared to T3/4 (*n* = 1/12, 8% ± 17%, *p*-value = 0.0026, Fig. 2c). Conversely, T3/4 tumors (*n* = 10/12, 78% ± 35%) gave rise to a significantly higher fraction of solid organoids compared to both Ta LG (*n* = 0/5, 8% ± 8%, *p*-value = 0.0001) and HG (*n* = 1/8, 31% ± 27%, *p*-value = 0.0256), whereas T2 tumors (*n* = 6/7, 61% ± 32%) compared to Ta LG (*p*-value = 0.0237). We then investigated the correlation between PDO morphology and concomitant carcinoma in situ (CIS) in the PT, an important feature of aggressive disease. 6 out of 22 tumors presented a concomitant CIS, 4 of which generated organoids with a predominant solid morphology (Table 1).

PDOs and corresponding PT were evaluated for the expression of basal (CD44, p63, cytokeratin (Ck) 5/6/14) and luminal (uroplakin II (UPKII), GATA3, Ck 8/20) markers. The PT phenotype characterized by the predominant expression of either luminal or basal markers was mostly observed in PDOs, with PDOs expressing both luminal and basal markers observed less frequently (Fig. 2d, e, Supplementary Figs. 5–11). The luminal markers Ck20 and GATA3 were mostly expressed in the cytosol and nucleus of the same cells, respectively. In 3 cases, PDOs were positive for both basal and luminal markers (Fig. 2d, Supplementary Figs. 6a, 7a). Furthermore, they presented basal cells (CD44^+^ or p63^+^) in the core and luminal cells (CK8^+^, Ck20^+^, or GATA3^+^) in the outer rim of organoids, suggesting an organized structure. In 6 cases, both basal and luminal markers could be observed in organoids’ outer rim (Supplementary Figs. 5a, 6b, 7b, 8a, 11a, b).

Marker expression was associated with PDO morphology. Solid PDOs were associated with the expression of CD44 and Ck5/6 compared to hollow (*p*-value <0.0001, *p*-value = 0.0015, respectively) and mixed (*p*-value = 0.0006, *p*-value = 0.0056, respectively) PDOs (Supplementary Fig. 12a). In addition, solid PDOs were associated with the expression of Ck14 compared to mixed organoids (*p*-value = 0.0054). On the other hand, hollow organoids were associated with the expression of GATA3 compared to solid (*p*-value <0.0001) and mixed PDOs (*p*-value = 0.0003) and with the expression of UPKII compared to solid PDOs (*p*-value = 0.0008, Supplementary Fig. 12b). Mixed PDOs, were instead associated with the expression of Ck20 compared to solid and hollow organoids (*p*-value <0.0001, Supplementary Fig. 12b). Mixed organoids were also associated with UPKII marker compared to solid organoids (*p*-value = 0.0003) and with CD44 expression compared to hollow organoids (*p*-value = 0.0305).

About the tumor stage, CD44 expression in organoids was significantly associated with T3 tumors compared to Ta and to T2 (*p*-value <0.0001, Supplementary Fig. 12c). In addition, Ck14 expression in PDOs was significantly associated with T1 tumors compared to Ta (*p*-value = 0.0010) and to T3/4 (*p*-value = 0.0360). By contrast, Ck20 was most highly expressed in organoids derived from Ta tumors (*p*-value <0.0001, Supplementary Fig. 12d). In parallel, GATA3 expression was significantly reduced in PDOs derived from T3/4 tumors compared to Ta (*p*-value = 0.0003) and to T2 (*p*-value = 0.0022), and Ck8 expression was significantly decreased in organoids from T3/4 tumors compared to all tumor stages (Ta: *p*-value <0.0001, T1: *p*-value = 0.0440, T2: *p*-value = 0.0004, Supplementary Fig. 12d).

### PDOs retain key genomic features of PTs

**Fig. 3:**
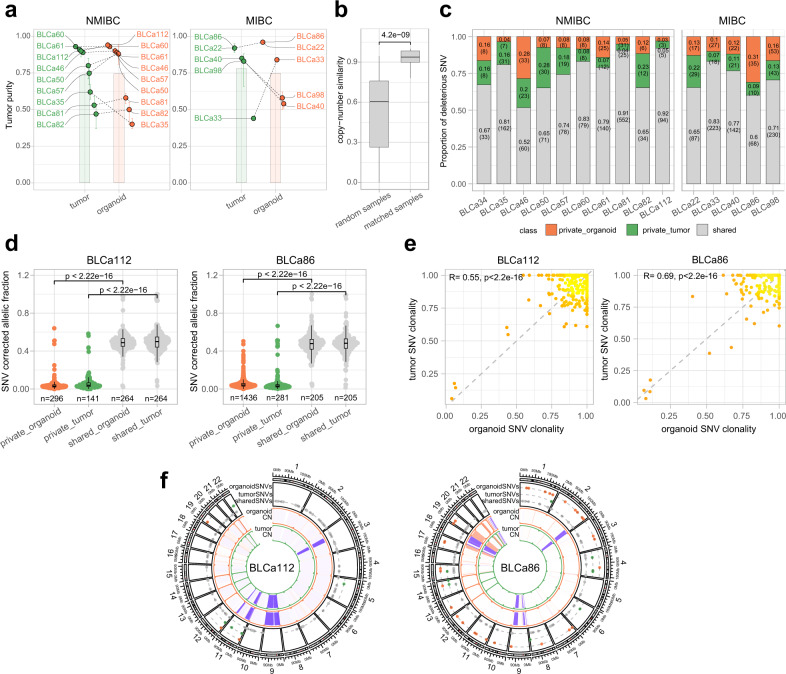
Patient-derived organoids (PDOs) retain key genomic features of parental tumors (PT). **a** Tumor purity in PT and matched PDOs of non-muscle invasive bladder cancer (NMIBC, left) and muscle invasive bladder cancer (MIBC, right) samples (*n* = 15 biological samples; mean estimate ± SE). Two-sided paired Wilcoxon test between PDOs and PT, *p*-value = 0.31. **b** Allele-specific copy-number (CN) similarity in randomly paired samples and matched paired ones (*n* = 312 randomly matched, *n* = 13 matched PDOs with corresponding PT). Boxplots indicate median (middle line), 25th, 75th percentile (box) and 5th and 95th percentile (whiskers). Two-sided Wilcoxon test, *p*-value = 4.2e^−09^. **c** Proportion of shared and private deleterious single nucleotide variants (SNVs) between PDOs and PTs. For each sample, proportions were compared using two-sided Chi-squared test, *p*-value <0.05. **d** Distributions of the tumor content and ploidy corrected allelic fraction (AF) of all the shared and private SNVs in PDOs and PTs in two representative samples (NMIBC BLCa112 left, *n* = 266 shared tumor SNVs, *n* = 263 shared organoids SNVs, *n* = 141 private tumor SNVs, *n* = 296 private organoid SNVs; MIBC BLCa86 right, *n* = 201 shared tumor SNVs, *n* = 209 shared organoids SNVs, *n* = 281 private tumor SNVs, *n* = 1436 private organoid SNVs). Boxplots indicate median (middle line), 25th, 75th percentile (box), and 5th and 95th percentile (whiskers). Two-sided Wilcoxon test, *p*-value <2.22e^−^^16^. **e** Clonality of shared point mutations of matched PDOs and PTs (BLCa112 and BLCa86). Two-sided correlation test *p*-value and Pearson’s correlation coefficient (R) are reported within the figure, *p*-value <2.22e^−16^. **f** Copy-number and point mutations profiles between PDOs and PTs for two representative samples (NMIBC BLCa112 left, MIBC BLCa86 right).

We determined the genomic landscape of PT and their corresponding PDOs. The tumor purity was comparable between PDOs and PTs (*p*-value = 0.31, Fig. 3a, Supplementary Data 2), with a trend for higher purity in organoids compared to matched PTs (12/15 pairs). No significant differences in tumor purity were detected between NMIBC and MIBC PTs (0.73 ± 0.18, 0.78 ± 0.22, respectively, *p*-value = 0.49) and PDOs (0.76 ± 0.24, 0.75 ± 0.20, respectively, *p*-value = 1).

By quantifying the similarity of copy-number profiles between two sequenced samples (“Methods”), we observed that matched PDO/PT pairs were significantly more similar than randomly paired samples from different patients (*p*-value = 4.2e^−09^, Fig. 3b). This is also evident based on the clustering analysis dendrogram (Supplementary Fig. 13a) that further presented a level of similarity determined by the polyploidy feature of a subset of tumors.

We further investigated the level of genomic concordance of matched PDO and PT samples based on deleterious point mutations. Across all sequenced samples, the mean fraction of shared mutations was 73% (±11.4%), whereas 12.5% (±8.1%) and 13.8% (±7.2%) of SNVs were exclusively detected in either the PDOs or the PT samples, respectively (Fig. 3c, Supplementary Fig. 13b).

When comparing the SNVs allelic fraction distributions, we observed significantly higher values for shared mutations (shared fraction) than for mutations found in only one sample of each pair (private fraction, Fig. 3d, Supplementary Fig. 14a). This result suggests that private mutations are only present in a small percentage of tumor cells. Nevertheless, several sub-clonal mutations were also preserved between PT and PDOs and their clonality profiles were significantly correlated (*p*-value <2.2e^−16^), hence suggesting that PDOs recapitulate the original tumor heterogeneity (Fig. 3e, Supplementary Fig. 14b). Side by side analysis of the genomes of paired samples suggests overall concordance (Fig. 3f).

We then investigated the correlation between PDO morphology and tumor purity, genomic burden (GB), and tumor mutational burden (TMB, see “Methods”). No significant tumor purity differences were observed in solid (0.64 ± 0.24), hollow (0.87 ± 0.17) and mixed organoids (0.78 ± 0.17, Supplementary Fig. 15a). From the genomic point of view, while TMB was not significantly different (solid: 4.23 ± 3.35, hollow: 7.97 ± 8.45, mixed: 10.31 ± 11.60), the GB was significantly higher in solid organoids (0.61 ± 0.37) than in hollow (0.06 ± 0.04; *p*-value = 0.0076) and mixed (0.14 ± 0.07, *p*-value = 0.0277) organoids, reflecting higher load of somatic-copy number aberrations and structural genomic alterations (Supplementary Fig. 15b, c).

### Tumors and their matched organoids recapitulate typical mutational mechanisms of BLCa

When comparing measurements of global genomic alterations of NMIBC (*n* = 10) and MIBC (*n* = 5) PDOs, we observed no significant difference in TMB (*p*-value = 0.486) and GB (*p*-value = 0.45). However, when stratifying NMIBC in LG and HG, we observed significant trends between the three ordered tumor classes (LG NMIBC, HG NMIBC, and MIBC) and TMB (Kendall Tau_B_ correlation = 0.42, *p*-value = 0.05) and GB (Kendall Tau_B_ correlation = 0.55, *p*-value = 0.03, Supplementary Fig. 16a, b). In particular, HG NMIBCs showed more features similar to MIBC PDOs than LG NMIBC. Conversely, tumor purity and allele-specific ploidy did not correlate with tumor stage and grade (Supplementary Fig. 16c, d).

**Fig. 4:**
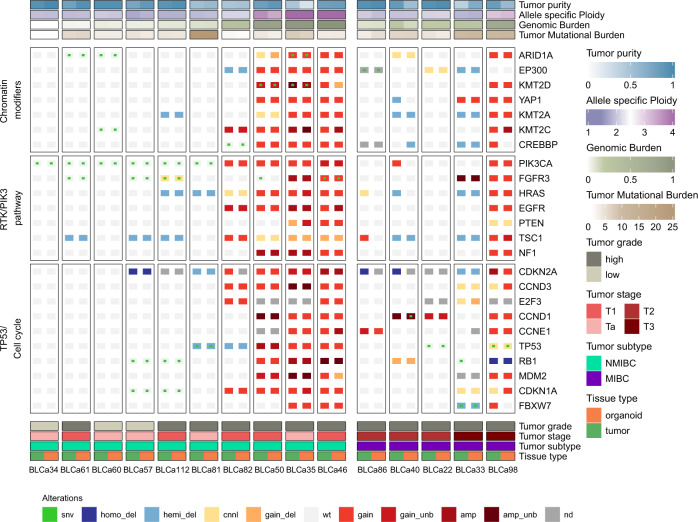
Tumor and matched patient-derived organoids (PDOs) recapitulate typical mutational mechanisms of bladder cancer (BLCa). Mutation heat-map. Samples are represented in the columns, with primary tumor on the left (green; *n* = 15 biological samples) and PDOs on the right (orange; *n* = 15 biological samples). PDO/PT pairs from the same samples are grouped by tumor subtype and ordered by increasing genomic burden within groups. The copy-number profile of the samples with high allele-specific Ploidy (asP, see “Methods”) is characterized by universal copy-number gains and amplifications. Rows represent genes grouped per pathway. Different types of genomic alterations are indicated in different colors in the bottom and Tumor purity, allele-specific Ploidy, Genomic Burden, and Tumor Mutational Burden are reported on the top. amp amplification, amp_unb amplification unbalanced, cnnl copy number neutral loss, gain_del gain deletion (gain with Loss of heterozygosity), gain_unb gain unbalanced, homo_del homo-deletion, hemi_del hemi-deletion, MIBC muscle-invasive BLCa, NMIBC non-muscle invasive BLCa, nd not determined, snv single nucleotide variant, wt wild-type.

Overall, our cohort of PT and matched PDOs recapitulated genomic alterations most commonly present in human BLCa (Fig. 4, Supplementary Data 3, 4). In line with previous findings^22^, we observed PIK3/AKT pathway genes to be frequently mutated in NMIBC with *PIK3CA* and *FGFR3* harboring deleterious point mutations in 60% and 50% of the cases, respectively (Fig. 4). *PIK3CA* and *FGFR3* mutations are considered important driving alterations that support NMIBC dysregulated growth^22^. No point mutations affecting *FGFR3* and *PIK3CA* genes were identified in MIBC samples (Fig. 4). *FGFR3* gene copy number gain instead was observed in 10% of NMIBC and 20% of MIBC cases, whereas *PIK3CA* gene copy number gain was observed in 40% of NMIBC and MIBC samples. Less prevalent mutations affecting the *PI3K/AKT* pathway, such as *EGFR* and *HRAS* gene alterations, could be identified in 30% NMIBC and 20% MIBC samples whereas mutations impacting *PTEN* gene were only observed in 20% of NMIBC samples.

According to the central role of tumor suppressor genes in BLCa progression^23^, alterations in genes with a key role in cell cycle regulation were more prevalent in the MIBC samples (Fig. 4). While 20% of NMIBC and MIBC samples were characterized with *MDM2* gene copy number gain or point mutations in *RB1* gene, 40% of MIBC samples and only 10% of NMIBC harbored deleterious *TP53* point mutations. Moreover, homo-deletion of the *RB1* gene and copy number gain of both *CCND1* and *CCNE1* genes occurred in 20% and 40% of MIBC cohort, respectively, but these mutations were not observed in NMIBC cohort. The *CDKN2A* gene was homo-deleted in 40% of MIBC and 10% of NMIBC samples and hemi-deleted in 20% and 10% samples, respectively.

Consistent with previous studies that define mutations affecting epigenetic regulators as early BLCa genomic alterations^22,23^, we found that chromatin-modifying genes were frequently mutated in both NMIBC and MIBC samples (Fig. 4). Among these, *ARID1A* gene was affected in 30% of NMIBC samples, whereas *KMT2A* gene was altered in 40% of MIBC samples.

We next performed an SNVs enrichment analysis by using genes harboring at least one deleterious point mutation in the PDOs. This resulted in 16 frequently enriched pathways across the cohort (see “Methods”, Supplementary Data 5). Some of these included signaling by FGFR1, FGFR3, and ERBB2 and chromatin organization and modifying enzymes. Mutations affecting the FGFR signature included genes involved in FGFR activation such as FGFs and genes downstream of the FGFR such as *MAPK1&3* and *BRAF*. The signaling by ERBB2 included mutations directly affecting ERBBRs such as *ERBB3&4* and downstream signaling mediators such as *AKT1, PIK3CA*, and *KRAS*. The signature of chromatin organization and modifying enzymes included genes relevant for BLCa, such as the *ARID1A* gene, which controls the gene transcription by modifying chromatin structure and the *KMT2D* gene, a histone methyltransferase.

### BLCa PDOs show heterogenous drug responses to SOC treatment

We next determined drug sensitivity profiles of early passage PDOs with a panel of FDA-approved drugs, including SOC drugs and compounds selected based on their clinical relevance, patient safety, and the possibility of drug repurposing. Compounds were deemed effective when significantly reducing organoid viability compared to vehicle (z-score below −1.5 and adjusted *p*-value <0.05).

**Fig. 5:**
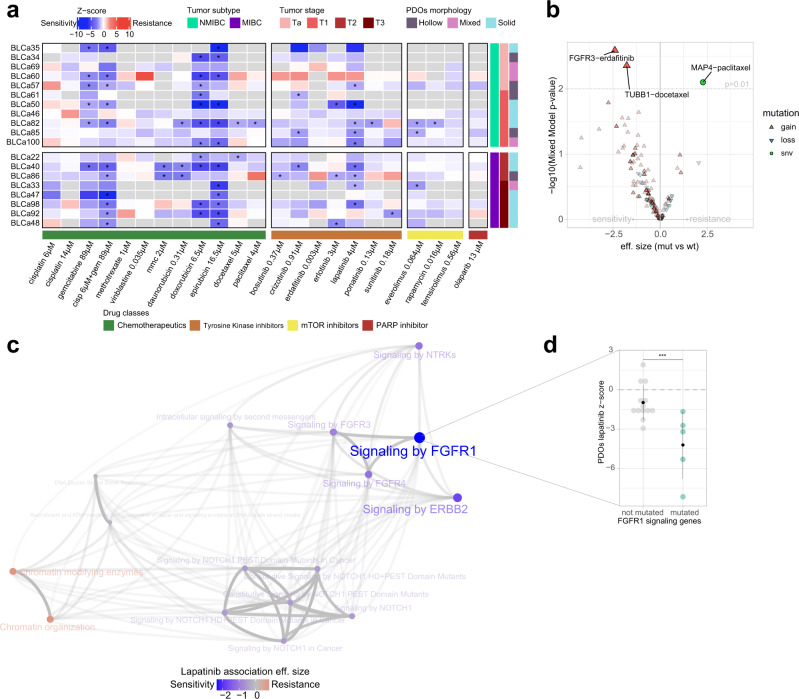
Patient-derived organoid (PDO) drug response and association with gene alterations. **a** Results of PDO drug screen assay (*n* = 19 biological samples). Heatmap reports the average of z-scores normalized to the vehicle values from cell viability assays after 48 h exposure of PDOs to drugs for one experiment for each biological sample (raw data are provided in Supplementary Data 6). Tumor subtype and stage, and PDO morphology are indicated in different colors on the right of the heatmap; not available data are in gray. Statistically significance between treatments and vehicle was calculated by one-way ANOVA test with Dunnet’s multiple comparison, *z-score ≤ −1.5 and adjusted *p*-value ≤ 0.05. **b** Genomic association analysis between genomic somatic events and treatment sensitivity/resistance. Reported *p*-value is obtained through Linear Mixed Model (LMM) fit (see “Methods”, section drugs association analyses) without adjusting for multiple comparisons. **c** Association analysis between frequently mutated pathways in PDOs and lapatinib sensitivity. Edges transparency encodes the proportion of shared genes between each term. Node size is proportional to the effect size of the association with lapatinib response (see “Methods”, section drugs association analyses). LLM. **d** Dot plot showing response to lapatinib in PDOs enriching for mutations on FGFR1 signaling genes compared to PDOs that do not show significant enrichment. Each data point corresponds to one biological sample (*n* = 18 for not mutated, *n* = 4 for mutated, mean +/− SD is reported in black) computed as the average z-score across technical replicates. *P*-value is obtained through LMM fit (see “Methods”, section drugs association analyses), ***False discovery rate = 0.02. cisp cisplatin, gem gemcitabine, mmc mitomycin C, mut mutation, wt wild type, snv single nucleotide variant.

Both NMIBC and MIBC PDOs showed heterogeneous responses to SOC drugs (Fig. 5a, Supplementary Data 6). While the SOC mitomycin C showed no significant effect in NMIBC PDOs, the SOC epirubicin was effective in 6 out of 10 samples (not pre-treated). Interestingly, one of the epirubicin-sensitive NMIBC samples (BLCa34) was derived from a patient who underwent post-resection treatment with epirubicin and did not experience any relapses (Table 2). BLCa69 and BLCa46 PDOs were not significantly sensitive to any of the tested drugs including SOC but showed an elevated response to cisplatin and gemcitabine (cisp/gem) combination and gemcitabine single treatment, respectively. In MIBC PDOs, the SOC cisp/gem combination was effective in 6 out of 7 samples (not pre-treated). Notably, one of the cisp/gem sensitive MIBC samples (BLCa48) was collected from a patient that underwent adjuvant chemotherapy (cisp/gem) and who currently is disease-free (Table 2). BLCa22 PDOs were not significantly sensitive to the SOC treatment but showed sensitivity to doxorubicin. In addition, PDOs from the BLCa33 sample, collected through a cystectomy post cisp/gem treatment, were not significantly sensitive to the combination and showed significant sensitivity to epirubicin (Table 2).

Among the additional tested chemotherapeutic drugs not used as first-line SOC, doxorubicin was effective to a high extent on both NMIBC and MIBC PDOs, and epirubicin on MIBC PDOs (Fig. 5a). Other drugs that were effective but to a smaller degree were daunorubicin, mitomycin C, docetaxel, and paclitaxel. Notably, daunorubicin was more effective on MIBC PDOs than on NMIBC PDOs, while mitomycin C was effective only on MIBC PDOs. Among the microtubule-targeting drug, docetaxel and paclitaxel were both comparably effective on NMIBC PDOs but not on MIBC PDOs, with docetaxel being more efficient. Cisplatin single treatment (low and high concentration), methotrexate, and vinblastine had no significant effect on either NMIBC or MIBC PDOs.

### PDO-based drug screening reveals sensitivity to targeted therapies

We used PDOs to screen targeted therapies. All broad-spectrum tyrosine kinase inhibitors (TKIs, i.e., bosutinib, crizotinib, and ponatinib) were effective in at least one sample. In contrast, only a subset of narrow spectrum TKIs elicited a significant response in PDOs (erlotinib, lapatinib, and sunitinib, Fig. 5a, Supplementary Data 6). TKIs targeting similar tyrosine kinases clustered together, showing highly correlated responses across different samples (Supplementary Fig. 17a). In particular, the effect of FGFR inhibitors ponatinib and erdafitinib was significantly positively correlated (Pearson correlation coefficient = 0.60, *p*-value = 0.08). Across the tested cohort of TKIs, lapatinib was the most effective treatment, significantly reducing organoid viability in 5 out of 10 NMIBC and in 4 out of 8 MIBC samples (Fig. 5a). Inter-drug comparisons showed that viability reduction induced by lapatinib was superior to the one induced by SOC on BLCa85 PDOs (lapatinib: 38% ± 14%, *p*-value = 0.0015; epirubicin: 25% ± 15%, *p*-value = 0.1243) and on BLCa98 PDOs (lapatinib: 42% ± 12%, *p*-value = 0.0046; cisp/gem: 28% ± 54, *p*-value = 0.0069). Of note, PDOs of the NMIBC sample BLCa61, which did not show a significant response to epirubicin and mitomycin C, were significantly sensitive to crizotinib (Fig. 5a).

Across the tested mTOR inhibitors, everolimus was the most effective drug, significantly reducing organoid viability in 2 out of 6 NMIBC samples and 1 out of 8 MIBC samples (Fig. 5a).

### Pharmacogenomic association analysis identifies response biomarkers in PDOs

The PDO genomic and drug sensitivity profiles comparison revealed significant associations between genomic alterations and drug response. Samples presenting a copy-gain of *FGFR3* or *TUBB1* genes were significantly more sensitive to erdafitinib (*p*-value = 0.0025) and docetaxel (*p*-value = 0.0044), respectively. Conversely, samples with deleterious mutations in the *MAP4* gene showed increased resistance to paclitaxel (*p*-value = 0.0079; Fig. 5b). Given the broad effectiveness of lapatinib and its potential role in personalized therapy, we investigated the association between lapatinib responses and biological pathways frequently mutated across the PDO cohort (Fig. 5c). Samples enriched for mutations in genes involved in FGFR1, FGFR3, FGFR4, ERBB2, and NTRKs signaling were positively associated with lapatinib response. However, at a false discovery rate (FDR) of 10%, only the association with the signature of FGFR1-mediated signaling was statistically significant (FDR = 0.02, Fig. 5d). Gene sets related to chromatin modification and organization were not significant and only mildly negatively associated with lapatinib response.

Finally, despite not observing a significant association between PDO morphology and drug sensitivity, we observed an association between tumor stage and drug sensitivity. PDOs derived from MIBC samples were significantly associated with a higher sensitivity to cisplatin 6 μM (*p*-value ≤ 0.001, Supplementary Fig. 17b, c).

### Clinical application of PDOs for personalized monitoring of tumor reoccurrence and in vitro drug sensitivity profiling

Due to a high reoccurrence rate, NMIBC patients are frequently subjected to multiple resections. Therefore, in our cohort, we included 2 patients (patients 1 and 2) from whom two samples were longitudinally collected during their clinical follow-up and analyzed in detail.

**Fig. 6:**
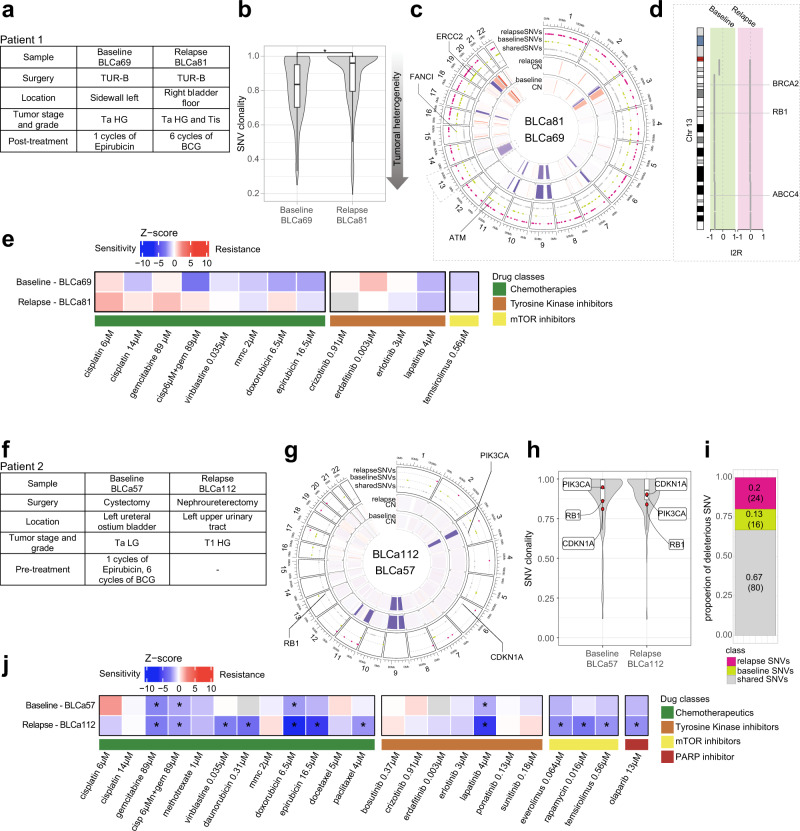
Monitoring tumor recurrence and progression and assessing drug sensitivity in vitro with longitudinally patient-derived organoids (PDOs). **a** Clinical and pathological information of patient 1. **b** Shared single nucleotide variant (SNV) clonality in PDOs baseline and relapse (*n* = 739 baseline SNVs; *n* = 607 relapse SNVs) and its associated gradient of tumoral heterogeneity (arrow pointing to higher tumoral heterogeneity). Box plots indicate median (middle line), 25th, 75th percentile (box), and 5th and 95th percentile (whiskers). Two-sided paired Wilcoxon test, *p-value <2.2e^−16^. **c** Copy-number (CN) and point mutations profiles between PDO from the baseline and the relapse. Relevant genetic alterations are highlighted. **e** Highlight on chromosome 13 deletion with affected genes. **d** Results of one PDO drug screen assay on patient 1. Heatmap represents the average of z-scores normalized to the vehicle values from cell viability assays after 48 h exposure of PDOs to drugs for one experiment. Samples code is indicated on the right side whereas tested drugs on the bottom of the heatmap. Not available data are in gray. One-way ANOVA with Dunnet’s multiple comparison test between treatments and vehicle (raw data are provided in Supplementary Data 6). **f** Clinical and pathological information of patient 2. **g** CN and point mutations profiles between PDOs from the baseline and the relapse of patients 2. Relevant genetic alterations are highlighted. **h** SNV clonality profiles of baseline PDOs and relapse PDOs with a highlight for *PI3KCA*, *RB1*, and *CDKN1A* genes. **i** Proportion of deleterious SNVs shared and not shared between baseline and relapse PDOs. **j** Results of one PDO drug screen assay on patient 2. One-way ANOVA with Dunnet’s multiple comparison test between treatments and vehicle, *z-score ≤ −1.5 and adjusted *p*-value ≤ 0.05 (raw data are provided in Supplementary Data 6). BCG Bacillus Calmette–Guérin, cisp cisplatin, Chr chromosome, Tis carcinoma in situ, gem gemcitabine, TUR-B transurethral resection of the bladder. HG high-grade, LG low-grade.

For patient 1*,* a baseline sample (baseline, BLCa69) was collected when the patient was treatment naive (Fig. 6a). Subsequently, the patient underwent local epirubicin treatment and experienced a first relapse after 46 days (Ta LG, unsampled), with no pharmacological treatment, and a second relapse after 197 days from baseline (relapse, BLCa81), followed by local BCG treatment (Fig. 6a, Table 2). The patient experienced further reoccurrences after the relapse sample (for details about the additional relapses see Table 2). Due to the frequent reoccurrences, the patient underwent cystoprostatectomy with the installation of an ileum conduit and did not experience further relapse. Patient 1 had chronic myeloid leukemia and was a former cigarette smoker.

Genomic characterization of PDOs from baseline and relapse from patient 1 showed a high TMB in both samples (baseline: 30.07, relapse: 24.84). The mean clonality of point mutations in the relapse sample was significantly higher compared to the baseline (*p*-value <2e^−16^, Fig. 6b) suggesting a reduction in cellular heterogeneity in the relapse. The SNVs that were not observed in the relapse (*n* = 215) were characterized by a significantly lower clonality, i.e., lost SNVs were more sub-clonal compared to the conserved ones (*p*-value = 0.00017, Supplementary Fig. 18a). In addition, the baseline PDOs were characterized by a large chromosome 13 hemi-deletion (Fig. 6c). This somatic copy number aberration spanned important genes involved in DNA damage repair (DDR) pathways such as *BRCA2* and *RB1*, as well as the *ABCC4* gene, a multidrug resistance-associated protein (Fig. 6d). Additionally, organoids from the baseline sample harbored an *ATM* loss-of-function point mutation (L1348F, Q2733*), a genetic alteration in the *FANCI* gene and a point mutation in *ERCC2* (E742K). Both baseline and relapse samples had impaired *TP53* gene (R280K).

Different compounds were tested on the PDOs from baseline and relapse of patient 1 (Fig. 6e, Supplementary Data 6). Interestingly, both baseline and relapse PDOs were not significantly sensitive to epirubicin, with the relapse PDOs being less sensitive than the baseline PDOs (z-score: baseline, −1.76 ± 0.3, relapse, −0.57 ± 0.45; nonparametric two-sided Wilcoxon test, *p*-value = 0.0571). Remarkably, PDO derived from relapse 2 and relapse 3 maintained epirubicin resistance (Supplementary Fig. 18b).

For what concerns patient 2, the baseline sample (BLCa57) was obtained from the cystectomy. Patient 2 was previously treated with epirubicin and 6 cycles of BCG and diagnosed with panurothelial disease (Fig. 6f). This patient experienced a relapse (BLCa112) in the left upper urinary tract, 488 days after the surgery. The baseline sample was diagnosed as Ta LG, whereas the relapse as T1 HG.

Genomic characterization of PDOs derived from Patient 2 showed that both samples harbored a mutation in the *CDKN1A* gene (Q29*), a target frequently mutated in BLCa and associated with tumor progression^24^, and in *RB1* gene (S624C, Fig. 6g). *CDKN2A*, a gene frequently associated with tumor progression, was homo-deleted in both samples (Figs. 4 and 6g). The high SNV clonality of these mutations was consistent in the two samples (Fig. 6h). In addition, baseline and relapse PDOs shared a high fraction of deleterious SNVs (*p*-value <0.05, Fig. 6i). Overall, these results suggest the conservation of key mutational events between baseline and relapse.

PDOs from the relapse (patient 2) sample were significantly more sensitive to epirubicin than baseline PDOs (z-score: baseline, −1.39 ± 0.68, relapse, −5.38 ± 0.12; nonparametric two-sided Wilcoxon test, *p*-value = 0.0286, Fig. 6j, Supplementary Data 6). This could be due to the local epirubicin treatment, while the reoccurrence manifested in a region likely spared from contact with the drug. Interestingly, PDOs from both specimens showed similar drug response profiles and were significantly sensitive to lapatinib, gemcitabine, cisp/gem combination, and doxorubicin. Given the high sensitivity to lapatinib and mTOR inhibition, we investigated the mutational status of genes associated with the PI3K-AKT-mTOR pathway. PDOs from both samples harbored a deleterious point mutation on *PIK3CA* gene (E545K), commonly observed in BLCa and resulting in the expression of a constitutively active PI3K^25^ (Fig. 6g). Moreover, both samples harbored a hemi-deletion of the *TSC1* gene, which forms a complex with *TSC2* and acts as a negative regulator of mTORC1 (Fig. 4).

## Discussion

In this study, we developed a highly reproducible protocol for generating BLCa PDOs covering a wide range of disease stages with the potential to be implemented in drug screening assay for precision medicine.

Overall, the performed characterization demonstrated how PDOs recapitulated the PT histopathological and molecular heterogeneity in terms of tumor purity, deleterious point mutations, and tumor cell sub-populations. PDO genomic profiles were highly comparable to the ones of PT and preserved peculiar BLCa alterations. Among these, alterations affecting the *FGFR3* gene in NMIBC or cell cycle regulators in MIBC emerged in our cohort^22,23^. The few discordant genomic features between matched PT and PDO might result from specific PT subclonal events or technical limitations in events detection (i.e., low limit of detection). However, the concordance between PDO and PT genomic profiles represents a key feature of PDOs, not only in bladder^12,26^ and upper urinary tract urothelial cancers (UTUC)^27^ but also in other cancer types^14,17–19,28^. In addition, this cohort of PDOs/PT pairs is well representative of BLCa clinical cases, with HG NMIBC PDOs showing more similar features to MIBC PDOs as compared to LG NMIBC PDOs, a feature already observed in the literature^9^. Furthermore, marker analyses at the scRNA and protein level confirmed that BLCa PDOs retained the main tumor phenotype.

The protocol used in this study was established by adapting growth conditions originally designed for prostate cancer organoids^21^. These included a growth factor-enriched medium in the absence of extracellular matrix support (ECM, Matrigel), intending to reduce biological variability, controlling stromal cells overgrowth, increasing drug accessibility, and reducing culture time, thus improving the clinical usability of this tool^21^. Furthermore, based on the observation that an ECM support could bias organoid cultures towards a basal phenotype^12,29^, we opted for an ECM-free culture to increase the likelihood of preserving both basal and luminal cells. In our approach, PDOs were used within the first passages. This methodology allowed us to create a clinically relevant model and maintain a high genomic and transcriptomic profiles similarity between PT and PDOs pairs.

PDOs from other cancers were already observed to display morphologies similar to the PTs^16,28,30^. In this work, BLCa PDOs grew according to diverse structures (i.e., solid, hollow, and mixed morphologies). Previous studies described an association between a hollow morphology with an enrichment of non-cancerous cells^27^; however, the high tumor purity confirmed a tumor origin for all three PDO morphologies described in this study. Therefore, PDO morphology may reflect tumor-specific features: compared to the other morphologies, solid organoids were mostly derived from high-stage and high-grade tumors characterized by concomitant CIS and associated with a high genomic burden. Furthermore, in line with previous analyses of transcriptomic profiling of MIBC^11,31^, solid organoids were associated with the expression of basal markers such as CD44 and Ck5/6 compared to hollow and mixed ones, mostly associated with luminal markers.

We demonstrated the clinical relevance and feasibility of using a BLCa PDO-based drug assay for comparing SOC drugs with other chemotherapeutic drugs. Response to SOC was highly heterogeneous with approximately 40% of NMIBC and 14% of MIBC PDOs showing no response. Chemotherapeutic agents that showed broad effectiveness on NMIBC PDOs included gemcitabine and doxorubicin. These results matched previous studies in which both compounds proved effective at reducing relapse and progression rates^32–35^. Conversely, cisplatin as single treatment showed low efficacy on NMIBC PDOs, but had a more prominent effect on MIBC PDOs. In the case of MIBC PDO, the drug screening provided additional chemotherapeutic drugs such as doxorubicin, epirubicin and mitomycin C. Overall, our findings recapitulate current treatment outcomes in clinically relevant settings and define PDOs as an attractive tool to stratify patients according to drug sensitivity profiles.

We have explored FDA-approved drugs targeting relevant and frequently altered pathways in BLCa such as the FGFR, the EGFR/ERBB2, and the mTOR pathways^36^. PDO sensitivity to targeted therapies was highly heterogeneous and, in a few cases, correlated with the sample-specific genomic background. Erdafitinib, an FGFR2/3 inhibitor approved as second-line for locally advanced or metastatic BLCa, was significantly more effective in samples harboring *FGFR3* copy number gain (BLCa46, BLCa35, BLCa98) or gene amplification (BLCa33). Among TKIs, we focused on lapatinib, which is of particular clinical interest given the frequent overexpression of its targets (*EGFR/ERBB2*) in BLCa^37^. Its clinical effectiveness however was less evident in previous studies, likely due to insufficient patient stratification or the limited predictive role of *EGFR/ERBB2* status^38^. Within the PDO cohort, samples enriching for mutations in the FGFR1 pathway were more sensitive to lapatinib. Although the association emerged by considering *the* FGFR1 pathway, mutated genes are also involved in numerous other similar pathways, including ERBB2. Finally, we also identified an association between *TUBB1* and *MAP4* genes mutations and sensitivity to docetaxel and paclitaxel, respectively. These drugs showed promising results in BLCa^39–41^.

In our study, the potential of PDOs in personalizing therapy is highlighted by the two retrospective longitudinal studies. In patient 1, PDOs were derived from two lesions before and after epirubicin treatment. The PDOs mimicked patient’s response and mirrored tumor evolution in vitro. Based on the decrease in SNV clonality and the loss of sub-clonal SNVs in the relapse compared to baseline, we hypothesized a drug-induced selection of a pre-existing, epirubicin-resistant population in the relapse PDOs supported by genetic alteration in key targets of multiple DDR pathways (*ATM*, *FANC1*, *ERCC2*, *BRCA2*, and *RB1*) detected in the baseline PDOs. Cancer clones bearing loss-of-function mutations in DDR pathways could have a higher susceptibility to anthracyclines and other DNA damage agents, as already observed in other studies^42–44^. It is also important to note that the PDOs from the baseline sample showed only a partial response to epirubicin, functionally supporting the existence of resistant clones already in the baseline sample.

On the other hand, the study involving patient 2, diagnosed with panurothelial disease, is relevant to highlight the application of PDOs to identify drug candidates for patients highly predisposed to tumor relapse. Furthermore, the high similarity between the baseline and the relapse samples, both in their genomic profile and in their in vitro responses suggests that the baseline sample could be informative for selecting a possible adjuvant therapy that could have been potentially effective in preventing the relapse. In this case, a possibly effective drug could be lapatinib, whose sensitivity could be supported by a mutation activating the PIK3-AKT pathway (*PIK3CA* E545K^45^).

In summary, with this study, we have generated a unique biobank resource of BLCa organoids that encompasses a broad spectrum of disease stages. Moreover, we have demonstrated that PDOs retain cancer heterogeneity and mutational burden and can be employed in drug-sensitive screens.

## Methods

### Patient clinical characteristics

All analyses were carried out in accordance with protocol approved by the Ethical Committee Bern (Cantonal Ethical approval KEK 06/03 and 2017-02295). Forty-nine bladder cancer samples and matching blood were collected from 38 patients undergoing transurethral resection of the bladder (TUR-B), cystectomies, or nephroureterectomy at the Inselspital, University Hospital in Bern.

Clinical details of the patients included in this study are reported in Tables 1, 2 and Supplementary Data 1. The patient cohort comprised 34 males and 4 females at the time of the sampling were 42 to 91 years of age (median of 69 years). In addition, a subgroup of 22 (N_NMIBC_ = 13, N_MIBC_ = 9) samples representative of the total patient cohort was selected for further analyses (i.e., genomic, marker expression, and drug screening analysis). Histopathological evaluation (performed by a certified pathologist) was performed on these samples group and are reported in Table 2.

### Sample collection

Tumor tissues from TUR-B, cystectomies, or nephroureterectomy from patients diagnosed with urothelial BLCa were collected in Dulbecco’s MEM media (Gibco, 61965-026) supplemented with 100 µg/ml Primocin (InVivoGen, ant-pm-1). In case of TUR-B, cold cup biopsies were used for tissue sampling and non-cauterized tissue was selected. For cystectomies and nephroureterectomy, tissue was sampled in the OR immediately after the bladder was harvested to reduce the tissue’s hypoxic damage. Either tumor samples were directly digested for organoid derivation or cryopreserved at −80 °C in Fetal Bovine Serum (FBS; Sigma, F7524) with 10% DMSO (Sigma, D2650). Blood was collected in RNA or EDTA blood tubes and stored directly at −80° or white blood cells (WBC) were cryopreserved in FBS/10%DMSO after lysation of erythrocytes in cold EC lysis buffer (150 mM NH_4_Cl, 10 mM KHCO_3_, 0.1 mM EDTA in dH_2_O).

### Tissue digestion and organoid derivation

BLCa tissue was collected in Basis medium (Advanced DMEM F12 Serum Free medium (ThermoFisher, 12634028) containing 2 mM GlutaMAX supplement (ThermoFisher, 35050061), 10 mM HEPES (ThermoFisher, 15630056) and 100 μg/ml Primocin). After mechanical disruption, tumor tissue was washed in Basis medium (200 g, 5 min) and digested in enzyme mix (5 mg/ml collagenase II (ThermoFisher 17101015) dissolved in Basis medium; 15 μg/ml DNase I (Roche, 10104159001) and 10 μM Y-27632-HCl Rock inhibitor (Selleckchem S1049)). Enzyme mix volume was adjusted so that tumor volume was no more than 1/10 of the total volume. Tissue was incubated at 37 °C for 1–2 h, mixing every 20 min. After digestion tissue was washed with Basis medium (400 g, 5 min). Pellet was incubated in 5 ml cold EC lysis buffer for 10 min at room temperature and then washed in an equal volume of Basis medium (400 g, 5 min). The cell pellet was suspended in 3–5 ml TrypLE Express (ThermoFisher, 12605028) depending on the pellet volume and incubated at 37 °C for 10–15 min with mixing every 5 min. Afterward cell suspension was passed through 50 μm cell strainer (CellTrics, 040042327) and the strainer was extensively washed with 5 ml TrypLE Express and Basis medium. Cells were washed in Basis medium (400 g, 5 min). The cell pellet was reconstituted in organoid medium and, after determine cell density, cells were seeded in ultra-low attachment (ULA) plates (Corning, 7341582). Generally, 300,000–500,000 cells were seeded per well in 6-well plates in 1–1.5 ml medium. Organoids were incubated at 37 °C with 5% CO_2_. BLCa organoid media contains the following reagents: Basis medium containing 5% FBS (Gibco, 1027-106), 1x B27 supplement (ThermoFisher, 17504044), 10 mM Nicotinamide (Sigma, N0636), 500 ng/ml R-Spondin (Peprotch, 120-38), 1.25 mM N-acetyl-cysteine (Sigma, A9165), 10 μM SB202190 (Selleckchem, S7067), 100 ng/ml Noggin (Peprotech, 25038), 10 ng/ml Wnt3a (Peprotech, 31520), 50 ng/ml hepatocyte growth factor (HGF; Peprotech, 10039), 500 nM A83-01 (Tocris, 2939), 50 ng/ml epidermal growth factor (EGF; Peprotech, AF-100-15), 10 ng/ml fibroblast growth factor 10 (FGF10; Peprotech, 100-26), 10 μM Y-27632 Rock inhibitor. Media was stored at 4 °C for no longer than 1 week and it was added to the culture every 3 days and completely changed after 1 week.

### DNA isolation from blood, organoids, and tissue samples

According to the manufacturer’s protocol, DNA was isolated from organoids and blood using the dNeasy Blood and Tissue kit (Qiagen, 69504). Snap-frozen tissue was homogenized in 160 μl PBS by stainless steel beads (Qiagen, 69989) in TissueLyser MM300 (Qiagen, Germany) at 20 Hz for 2 × 2 min. The lysate was centrifuged at 12,000 × *g* for 10 min and the supernatant was collected for DNA isolation. Subsequently, DNA from tissue samples was extracted with ReliaPrem^TM^ gDNA Tissue Miniprep System (Promega, A2051) according to the manufacturer’s protocol. DNA concentration was assessed with Qubit dsDNA high-sensitivity or broad-range kits (ThermoFIsher, Q33233 and Q33263).

### DNA sequencing

#### Library preparation

Genomic DNA for library preparation is fragmented with Covaris M220 to a target size of 180–220 bp. Libraries for whole exome sequencing are prepared starting from 100 ng gDNA (for the sample derived from FFPE tissue the starting input is 90 ng) with Roche KAPA HyperPrep Kit following the SeqCap EZ HyperCap v2.3 protocol. For the hybridization with Roche SeqCap EZ Human Exome v3.0, up to 11 gDNA samples are multiplexed together mixing 100–200 ng of each library to obtain a combined mass of 1000–1100 μg and then incubated for capture at 47 °C for 16–20 h. Pre- and post-capture libraries are quantified using Qubit dsDNA High Sensitivity Assay and the quality is assessed with Agilent Bioanalyzer High Sensitivity DNA Kit. Samples were then sequenced Illumina NovaSeq 6000 paired-end, 150 bp.

### DNA sequencing analysis

#### Genomic analysis pipeline

FASTA files were trimmed using Trimmomatic^46^, quality checks were performed with FastQC^47^, and reads were aligned with BWA algorithm^48^ on hg38. Deduplication, realignment around indels and base recalibration were then performed using GATK4^49^. Finally, mutation, copy-number data, and samples level statistics were obtained through the recently established SPICE analysis pipeline^50^. Briefly, it includes quality control step to assess the similarity between matched samples by running SPIA^51^, allele-specific copy number assessment upon data segmentation by running CLONET v2^52^ and mutation and annotation calling via MuTect2^53^ and VEP^54^. Sequencing statistics from the pipeline as well as mean coverage are reported in Supplementary Data 7.

#### Allele-specific copy-number data analysis

Allele-specific copy-number data analysis was performed on 32 samples, 16 PDO and 16 tissue samples (Supplementary Data 2) for which data signal was deemed amenable by CLONETv2.

For each sample, we computed the following previously reported^50,55^ copy-number-based genomic indexes:

##### Allele-specific Ploidy (asP)^50^

asP is a measure proportional to the average amount of DNA per cell. Considering a set of copy-number segments $s$ of a genome $G$, the allele-specific Ploidy is defined as the weighted mean of allele-specific copy-number levels per segment:

$${asP}\left(G\right)=\,\frac{{\sum }_{s\in G}({cnA}(s)+{cnB}(s))\times {{{{{\rm{ws}}}}}}}{{\sum }_{s\in G}{{{{{\rm{ws}}}}}}}$$

with ${ws}$ being a segment length, ${cnA}(s)$ and ${cnB}(s)$ being copy-number states of each allele in segment $s$, with ${cnA}(s) > {cnB}(s)$ by definition.

##### Genomic Burden (GB)^55^

GB is defined as the proportion of the genome that is not wild-type (i.e., number of alleles different from 2). By design, triploid and tetraploid cells have genomic burden equal to 1.

We leveraged discrete copy-number levels of each allele, gene-wise, to compute a copy-number-based similarity score between two samples ${k}_{1}$ and ${k}_{2}$ (Fig. 3b, Supplementary Fig. 13a). The score is computed starting from: ${d}_{{k}_{1},{k}_{2}}={||}({{{{{\rm{cn}}}}}}{{{{{{\rm{A}}}}}}}_{{{{{{{\rm{k}}}}}}}_{1}},{{{{{\rm{cn}}}}}}{{{{{{\rm{B}}}}}}}_{{{{{{{\rm{k}}}}}}}_{1}})-({{{{{\rm{cn}}}}}}{{{{{{\rm{A}}}}}}}_{{{{{{{\rm{k}}}}}}}_{2}},{{{{{\rm{cn}}}}}}{{{{{{\rm{B}}}}}}}_{{{{{{{\rm{k}}}}}}}_{2}}){||}$ with $({{{{{\rm{cnA}}}}}},{{{{{\rm{cnB}}}}}})$ being the concatenated copy-number profiles of, respectively, major and minor alleles of a sample for each gene and |$\left|\,{{{{{\rm{X}}}}}}-{{{{{\rm{Y}}}}}}\,\right|$| the notation for the Euclidean distance of two vectors ${{{{{\rm{X}}}}}}$ and ${{{{{\rm{Y}}}}}}$. The computed pairwise distances are then normalized to obtain a rescaled pairwise-distance vector: ${{{{{{\rm{d}}}}}}}^{{\prime} }=\,\frac{d-{{{{{\rm{min }}}}}}(d)}{{{{{{\rm{max }}}}}}\left(d\right)-{{{{{\rm{min }}}}}}(d)}$ . Final similarity between samples ${k}_{1}$ and ${k}_{2}$ is then obtained as: ${s}_{{k}_{1},{k}_{2}}=1-{{d}^{{\prime} }}_{{k}_{1},{k}_{2}}$.

#### Deleterious SNVs and SNVs enrichment analysis

First, high-quality SNVs were selected by adopting the following filters: minimum tumor coverage ≥20, tumor allelic fraction ≥0.08, number of tumor alternative reads ≥5, and number of normal alternative reads = 0. By leveraging filtered SNVs for each sample, we computed *Tumor Mutational Burden (TMB)* as the total number of high-quality SNVs per sequenced DNA million-bases (Mb).

SNVs were then considered deleterious if their impact on protein function was annotated as medium or high by VEP^54^. Chi-squared test was used between number of shared and private SNVs in each sample. Wilcoxon-test instead was used between the allelic fraction of private and shared SNVs in each sample.

To perform the SNVs enrichment analysis, we selected all genes harboring at least one deleterious point mutation and performed an over-representation analysis exploiting Reactome Db gene sets collection^56^. For each tested PDO, we selected a subset of enriched terms (Q-value <0.2) and retained those shortlisted in at least 20% of the study samples (relapse samples BLCa112 and BLCa81 excluded).

### Drug screen assay

Organoids at p1 were collected and washed in basis medium (100 G, 5 min) and dissociated into single cells with 1 ml TrypLE Express at 37 °C for 10 min. Single cell suspension was counted, washed once in basis medium (100 g, 5 min), and resuspended in BLCa organoid medium. Cells were then seeded as replicates based on biological material available in ULA 384 well plate (Corning, cat. No. 4588) in 20 μl of BLCa organoids medium at 8000–10,000 cells per well. Generally, an average of 7 technical replicates for untreated and vehicles and 3 replicates at least for each drug condition were seeded (see Supplementary Data 6 for per-replicate data). After 48 h of culture, 20 μl of 2x drugs solutions or vehicle, diluted in complete BLCa medium, were added to organoid cultures. After 48 h of drug treatment, CellTiter-Glo 3D assay (Promega, G9682) was used to measure cell viability, following manufacturer’s indications with minor modifications. Shortly, 40 μl of CellTiter-Glo 3D reagent was added per well to the assay plates. Plates were subsequently shaken for 5 min and incubated at 37 °C for 25 min. After incubation, luminescence was measured using Tecan M200 Pro plate reader. Raw counts were normalized independently for each screened sample using the following formula: $\frac{{X}_{s}-{X}_{v}}{{{{{{{\rm{SD}}}}}}}_{{{{{{\rm{v}}}}}}}}$, ${X}_{s}$: technical replicates for each drug treatment; ${X}_{v}$: mean of technical replicates of the matching vehicle conditions; ${{{{{{\rm{SD}}}}}}}_{{{{{{\rm{v}}}}}}}$: standard deviation of the technical replicates of the matching vehicle conditions (Supplementary Data 6). Z-scores of cisplatin, gemcitabine as well as their combinations were generated using H_2_O as vehicle, while z-scores of all remaining drugs were generated using DMSO as vehicle. Raw counts were also used to generate fold-changes with respect to the average of vehicle raw values. From fold-change values the actual cell growth inhibition was calculated for each condition as 1 – fold-change.

### Reporting summary

Further information on research design is available in the Nature Portfolio Reporting Summary linked to this article.

## Supplementary information

Supplementary Information

Reporting Summary

Description supplementary files

Supplementary Data 1-7

## Data Availability

All unique materials are readily available upon request to the corresponding author. The whole exome sequencing data generated in this study have been deposited in the dbGAP database under accession code phs003149.v1.p1 Individual-level data are available for download by authorized investigators (https://view.ncbi.nlm.nih.gov/dbgap-controlled). Data dictionaries and variable summaries are available on the dbGaP FTP site (https://ftp.ncbi.nlm.nih.gov/dbgap/studies/phs003149/phs003149.v1.p1). The public summary-level phenotype data may be browsed at the dbGaP study report page (https://www.ncbi.nlm.nih.gov/projects/gap/cgi-bin/study.cgi?study_id=phs003149.v1.p1). Please refer to the release notes for more details (https://ftp.ncbi.nlm.nih.gov/dbgap/studies/phs003149/phs003149.v1.p1/release_notes/Release_Notes.phs003149.BladderCancerOrganoids.v1.p1.MULTI.pdf). Allele-specific copy-number and SNVs calls derived from Whole Exome Sequencing are available on GitHub (https://github.com/demichelislab/BLCa_organoids_data). Single-cells RNAseq generated in this study have been deposited in Gene Expression Omnibus (GEO) and is available under the accession number GSE217956. In addition, allele-specific TCGA data of MIBC BLCA patients used in this paper were obtained from (https://zenodo.org/record/5266542#.Y97dDi-B1sE) and MSK genomics data of NMIBC patients were downloaded from cBioPortal (https://www.cbioportal.org). Source data are provided with this paper.

## Source data

Source Data
