# Supplementary material for: Bladder cancer organoids as a functional system to model different disease stages and therapy response
Source: Nat Commun. 2023 Apr 18;14:2214. doi: 10.1038/s41467-023-37696-2 (PMC10113240; doi:10.1038/s41467-023-37696-2)
Supplement: Supplementary file 1 — Supplementary Information [file 41467_2023_37696_MOESM1_ESM.pdf]

Supplementary Information (Supplementary Figures)

Bladder cancer organoids as a functional system to model different disease stages and therapy response

Martina Minoli * and Thomas Cantore*, et al.

Supplementary Fig. 1

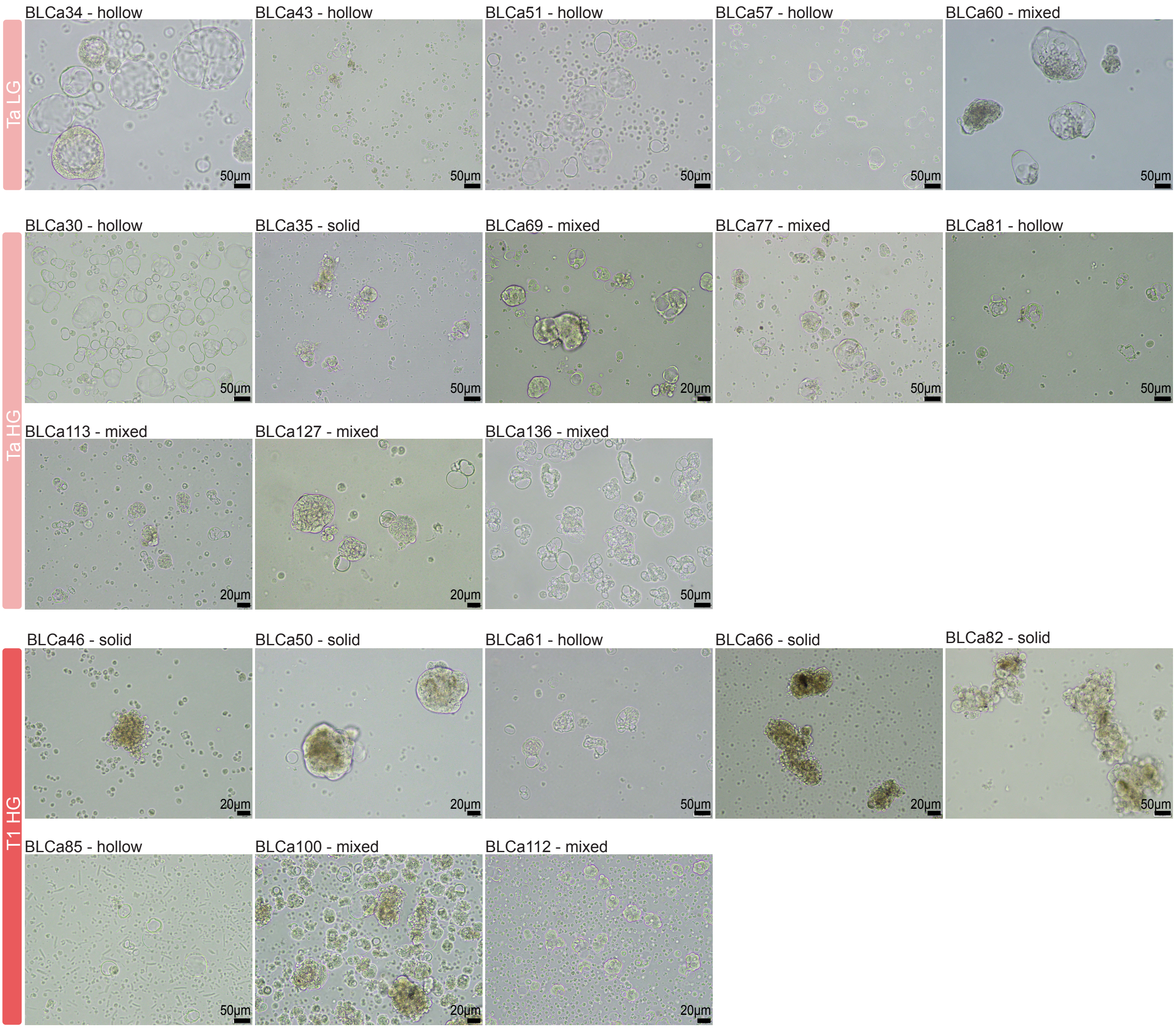

Supplementary Figure 1. Patient-derived organoids (PDOs) from non-muscle invasive bladder cancer (NMIBC) samples, related to figure 1.

Representative brightfield images of 21 NMIBC PDOs at passage 1 ordered by tumor stage and grade. The main PDO morphology and sample ID are reported on the top of the image (6 days of culture for BLCa60; 7 days for BLCa30, BLCa34, BLCa51, BLCa57, BLCa35, BLCa66, BLCa69, BLCa77, BLCa81, BLCa100, BLCa112, and BLCa113; 9 days for BLCa50, BLCa127, and BLCa136; 10 days for BLCa43 and BLCa82; 11 days for BLCa85, 13 days for BLCa61 and 14 days for BLCa46). LG, low-grade; HG, high-grade.

Supplementary Fig. 2

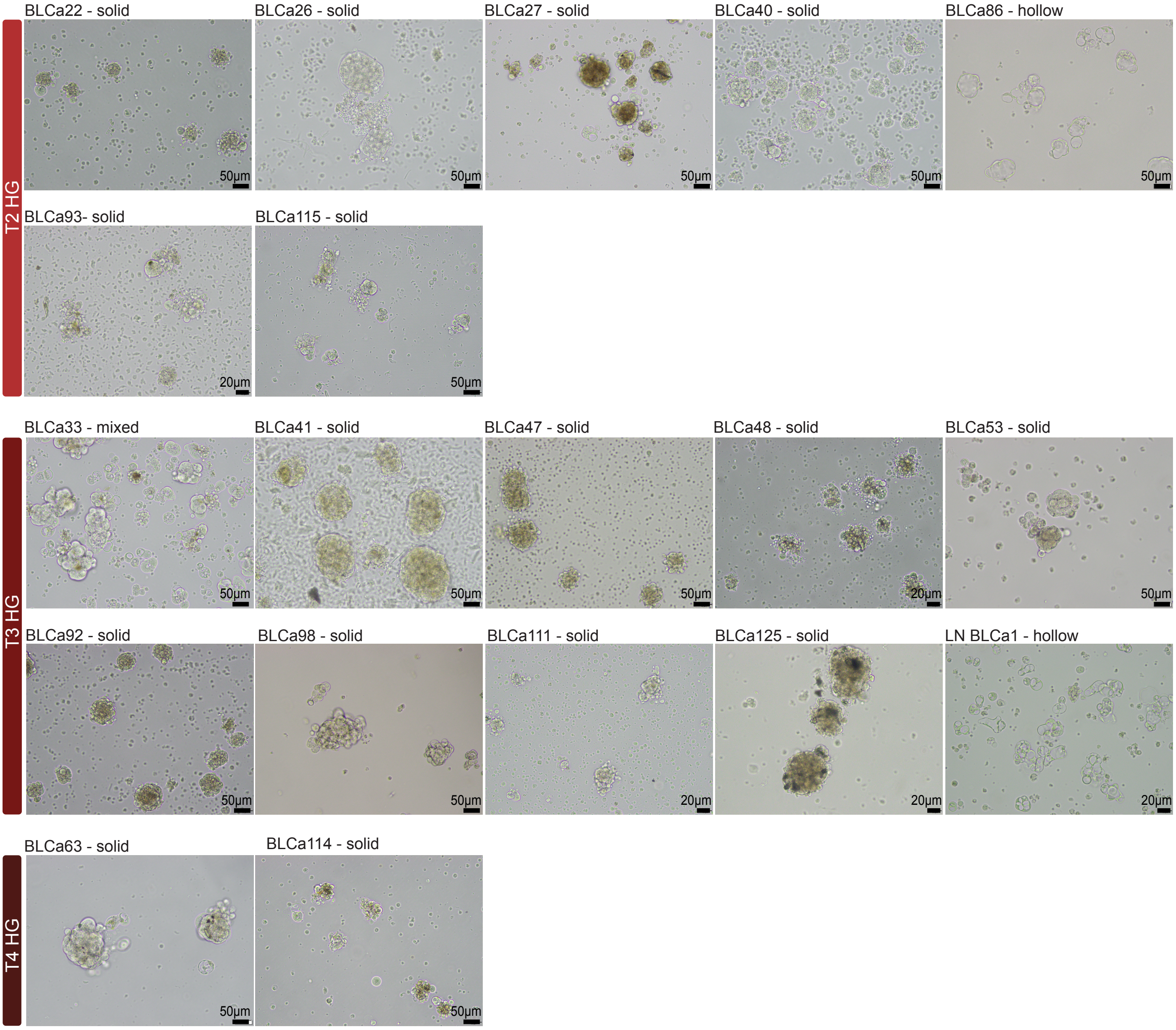

Supplementary Figure 2. Patient-derived organoids (PDOs) from muscle invasive bladder cancer (MIBC) samples, related to figure 1.

Representative brightfield images of 19 MIBC PDOs at passage 1 ordered by tumor stage and grade. The main PDO morphology and sample ID are reported on the top of the image (4 days of culture for LN_BLCa1; 5 days for BLCa47, and BLCa98; 6 days for BLCa93, and BLCa48; 7 days for BLCa86, and BLCa92; 8 days for BLCa22, and BLCa125; 9 days for BLCa40, BLCa115, and BLCa114; 10 days for BLCa33; 11 days for BLCa63, and BLCa111; 13 days for BLCa53; 14 days for BLCa27, and BLCa41; and 20 days for BLCa27). LG, low-grade; HG, high-grade.

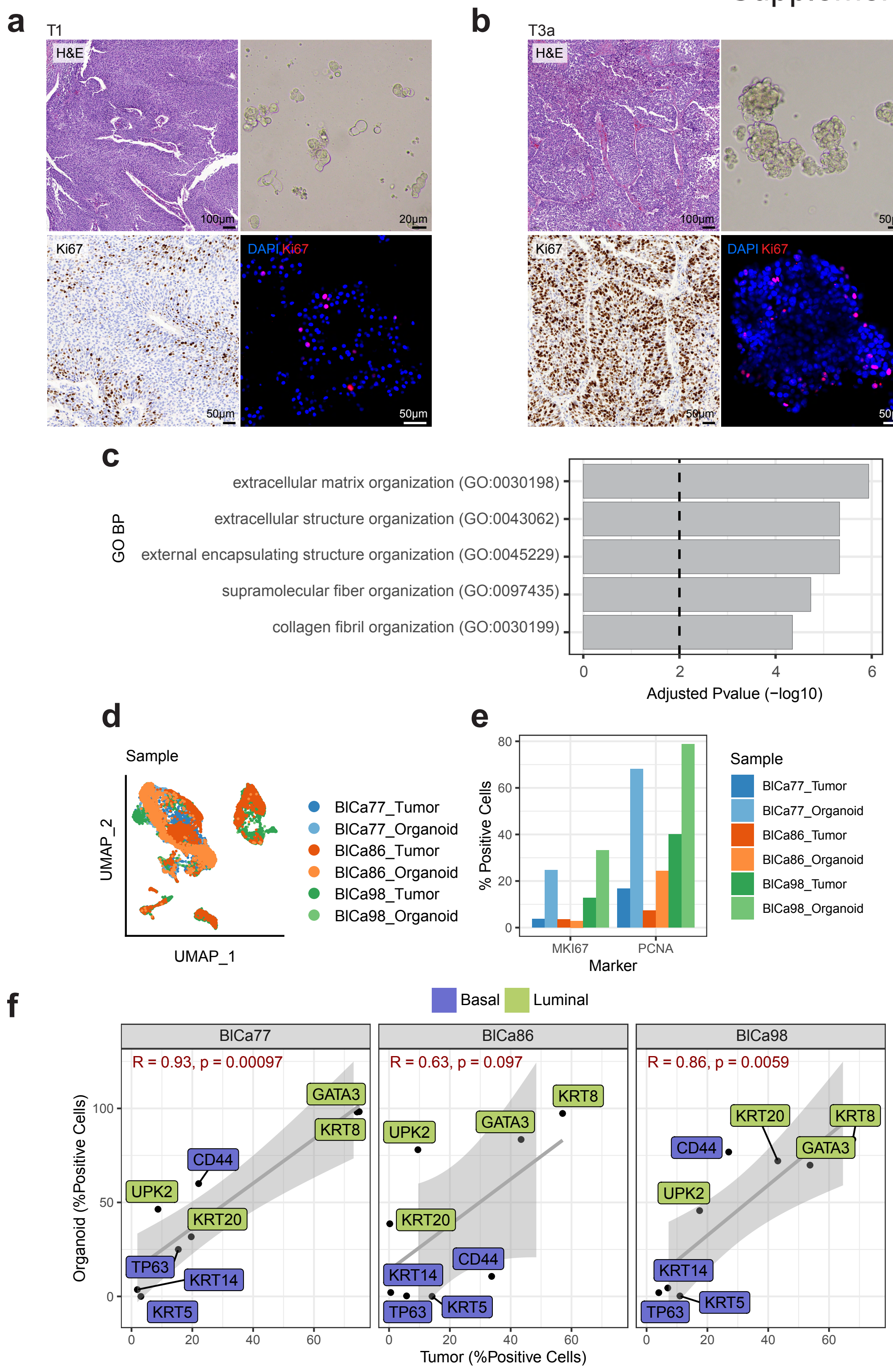

Supplementary Figure 3. Patient-derived organoids (PDOs) and parental tumor (PT) morphology and single-cell RNA analysis of PT/PDOs pairs, related to figure 1.

a-b Morphology of PT (Hematoxylin and Eosin staining) and PDOs (brightfield image, upper) at passage 1 for two representative cases (BLCa112, nephroureterectomy, T1 stage, (**a**) and BLCa98, cystectomy, T3a stage, (**b**)). Immunohistochemistry for Ki67 for PT, and whole-mount immunofluorescence staining for Ki67 for PDOs (bottom). **c** Gene Ontology (GO) Biological Process (BP) analysis for genes upregulated in tumor vs organoid. One-sided Fisher exact test adjusted with Benjamini-Hochberg method. **d** UMAP plot of cells derived from PT/PDOs pairs clustered by sample types. **e** Barplot of the fraction of positive cells for Ki67 and PCNA in PT/PDOs pairs. **f** Correlation of the fraction of cells positive for basal (blue) and luminal (green) markers in PT/PDOs pairs. Two-sided Pearson Correlation (R). BLCa, bladder cancer. KRT, cytokeratin.

Supplementary Fig. 4

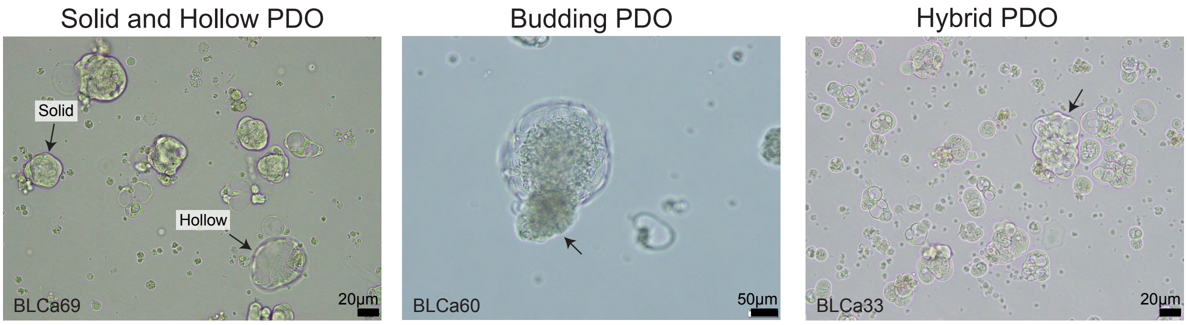

Supplementary Figure 4. Bladder cancer (BLCa) patient-derived organoids (PDOs) at passage 1 characterized with a mixed morphology, related to figure 2.

Representative brightfield images of BLCa69 sample (day 7) presenting solid and hollow organoids, BLCa60 PDOs (day 6) with budding structures, and BLCa33 PDOs (day 10) presenting a mix of solid and hollow features.

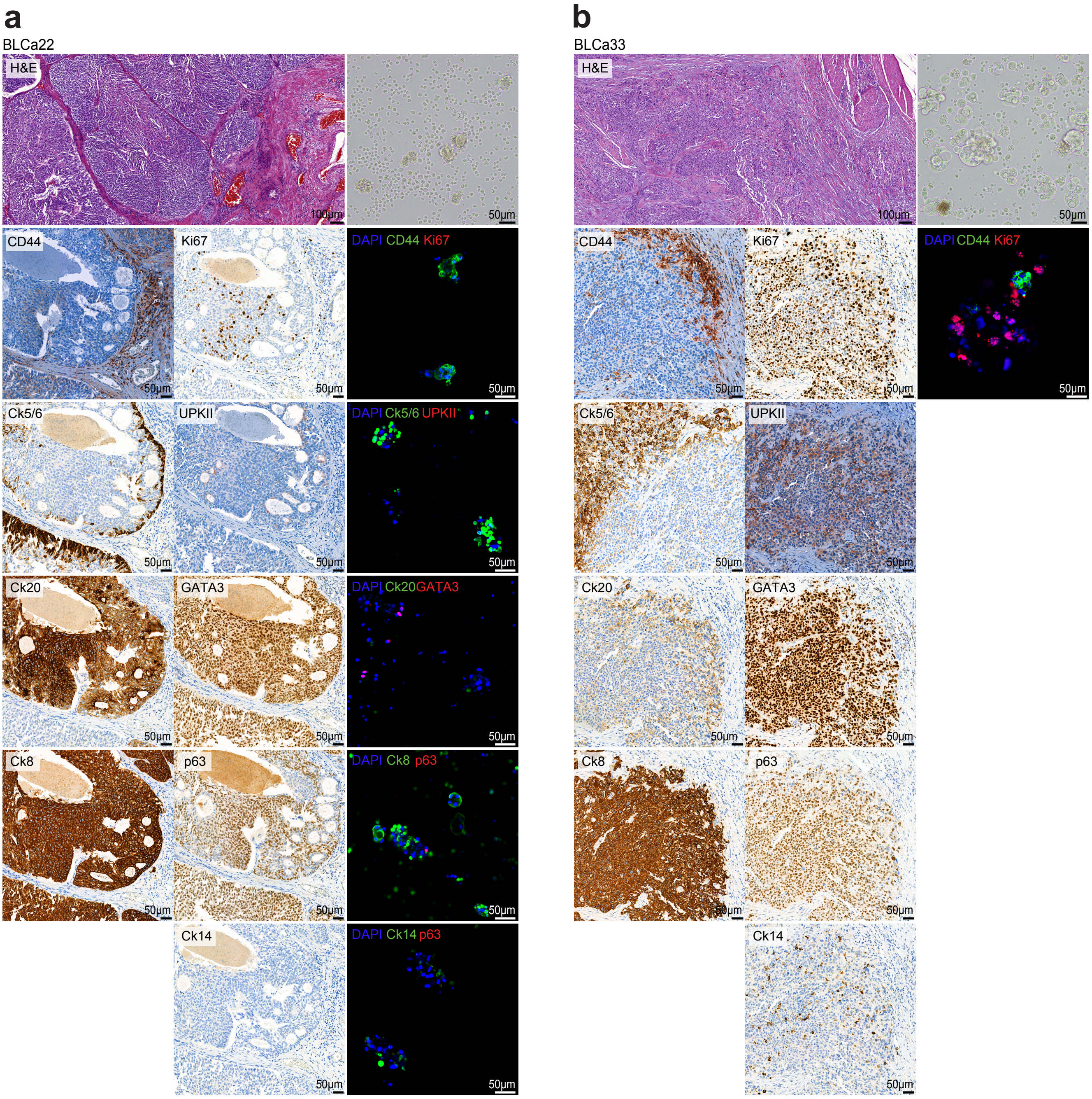

Supplementary Figure 5. Comparison of marker expression between organoids and parental tumor, related to figure 2.

a-b Hematoxylin and Eosin staining and immunohistochemistry staining of parental tumor for indicated markers and brightfield images and whole mount immunofluorescence staining of organoids at passage 1 for indicated markers. BLCa22 (**a**) and BLCa33 (**b**) samples.

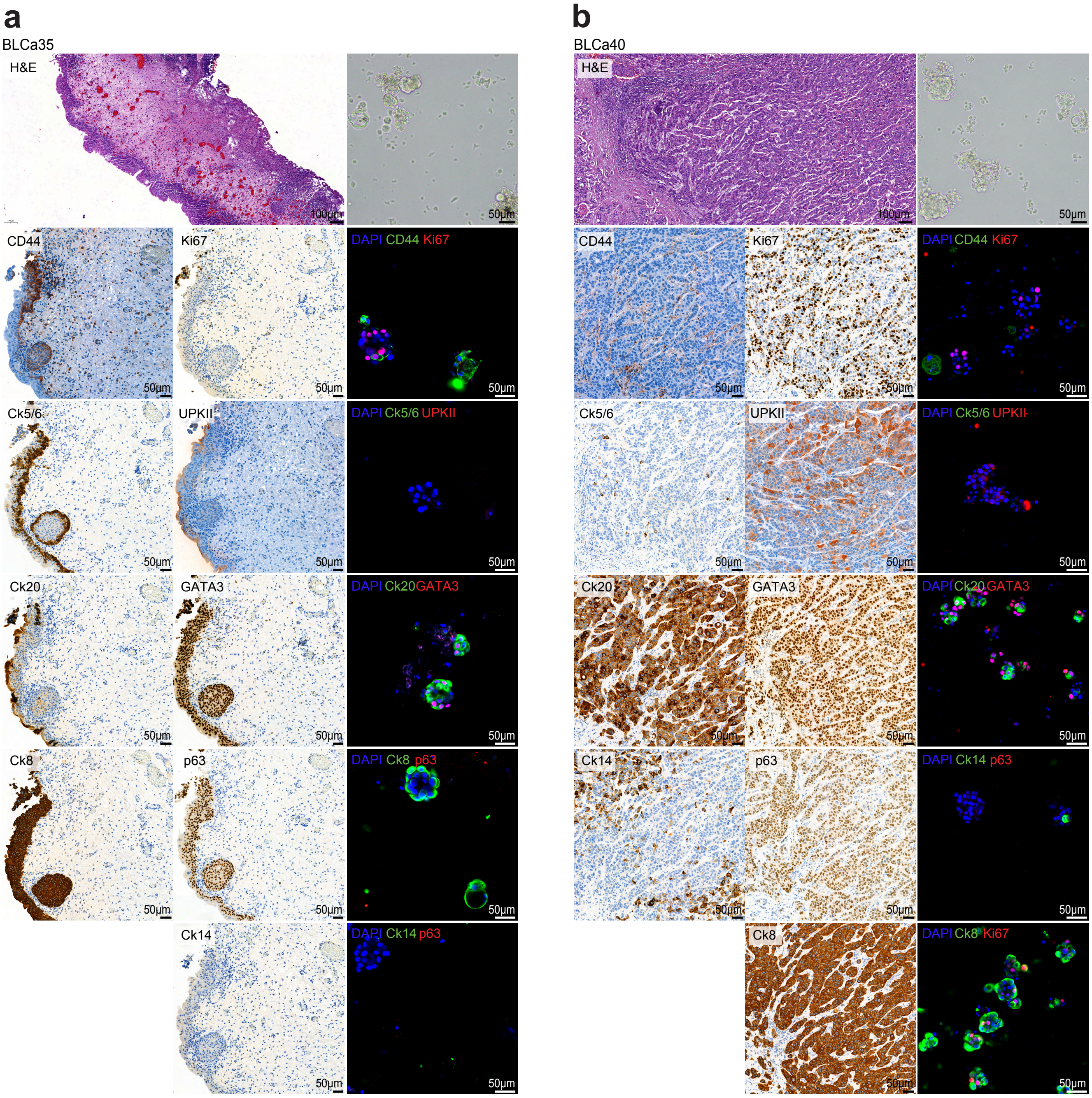

Supplementary Figure 6. Comparison of marker expression between organoids and parental tumor, related to figure 2.

a-b Hematoxylin and Eosin staining and immunohistochemistry staining of parental tumor for indicated markers and brightfield images and whole mount immunofluorescence staining of organoids at passage 1 for indicated markers. BLCa35 (**a**) and BLCa40 (**b**) samples.

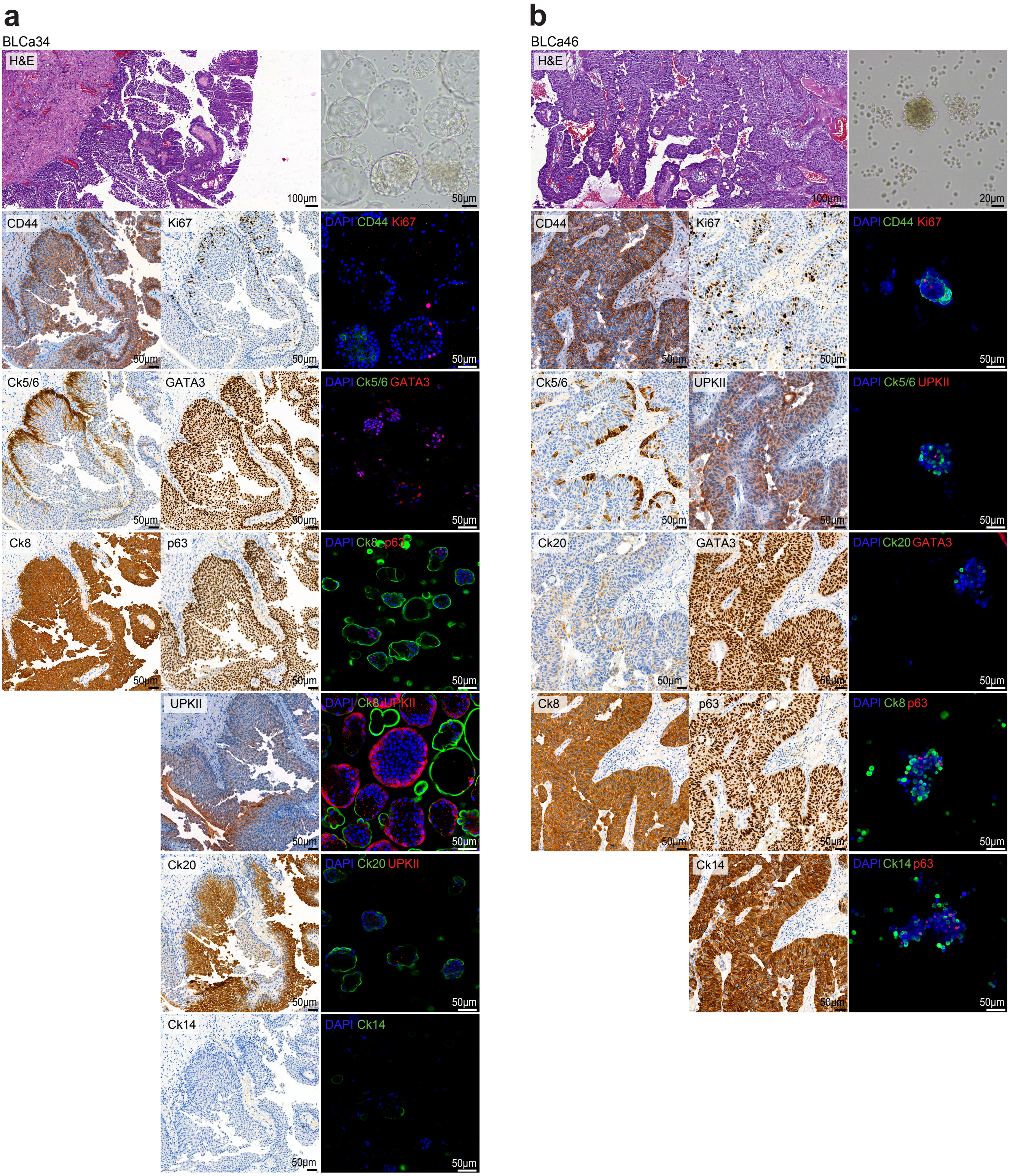

Supplementary Figure 7. Comparison of marker expression between organoids and parental tumor, related to figure 2.

a-b Hematoxylin and Eosin staining and immunohistochemistry staining of parental tumor for indicated markers and brightfield images and whole mount immunofluorescence staining of organoids at passage 1 for indicated markers. BLCa34 (**a**) and BLCa46 (**b**) samples.

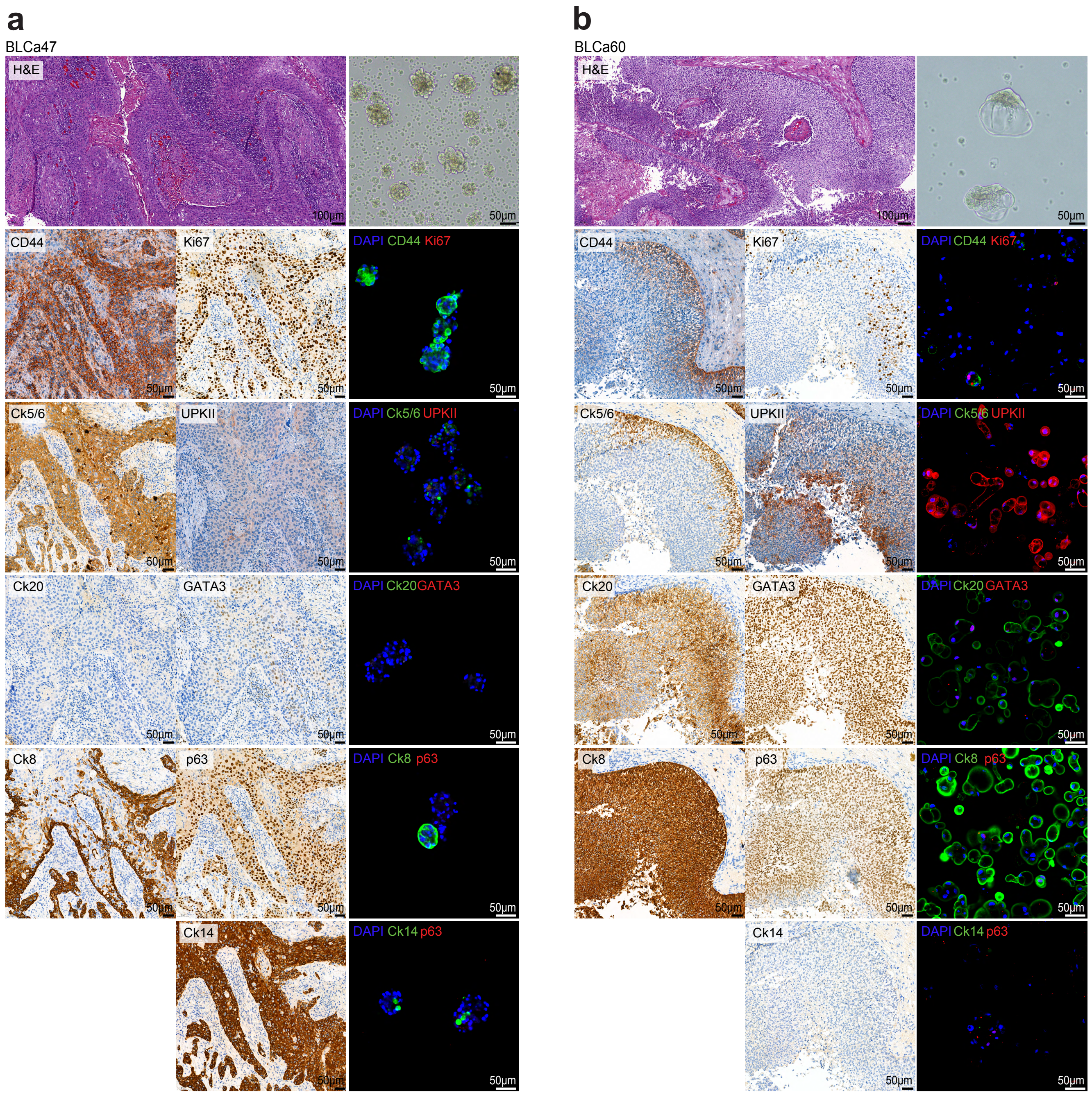

Supplementary Figure 8. Comparison of marker expression between organoids and parental tumor, related to figure 2.

a-b Hematoxylin and Eosin staining and immunohistochemistry staining of parental tumor for indicated markers and brightfield images and whole mount immunofluorescence staining of organoids at passage 1 for indicated markers. BLCa47 (**a**) and BLCa60 (**b**) samples.

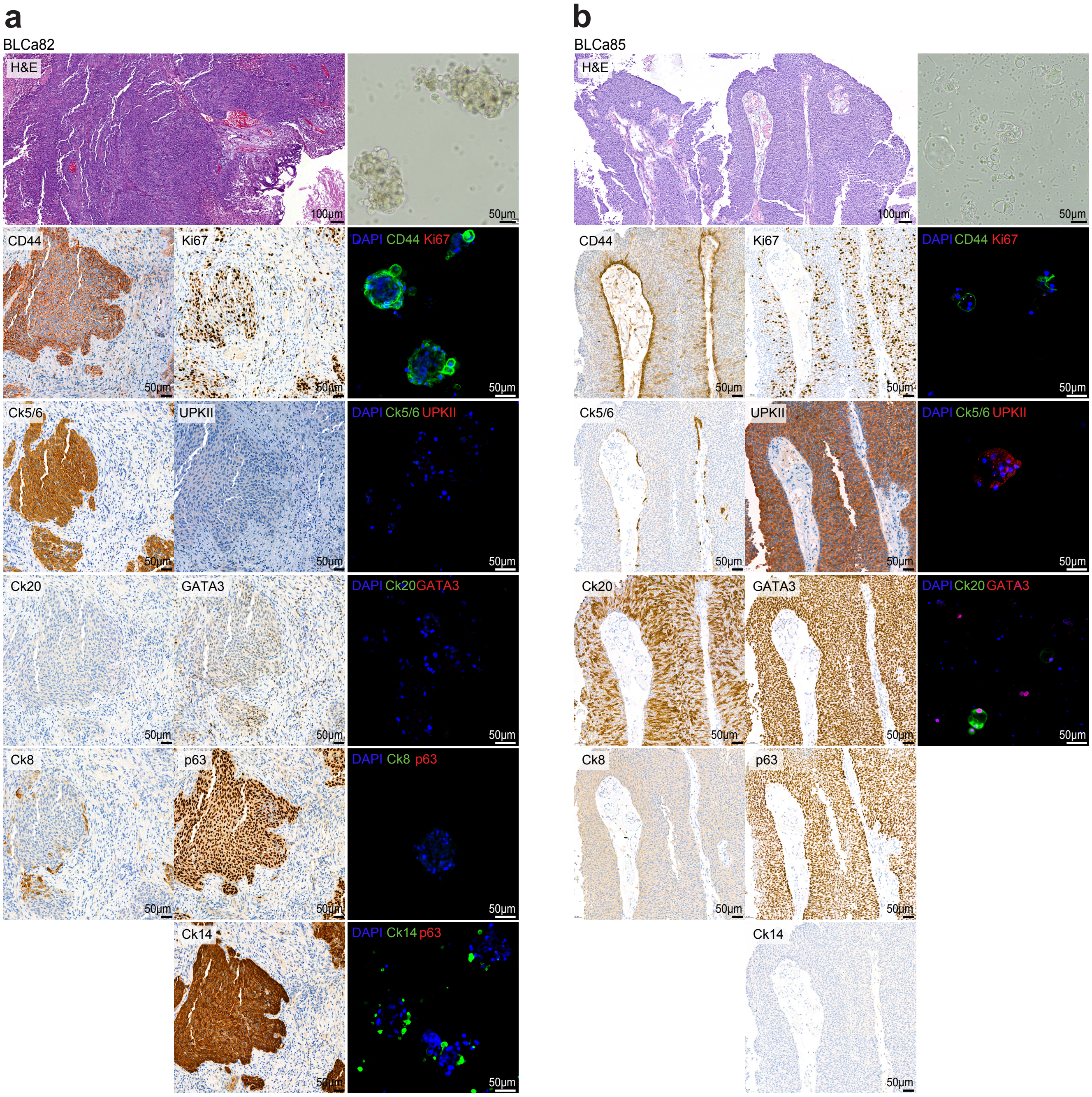

Supplementary Figure 9. Comparison of marker expression between organoids and parental tumor, related to figure 2.

a-b Hematoxylin and Eosin staining and immunohistochemistry staining of parental tumor for indicated markers and brightfield images and whole mount immunofluorescence staining of organoids at passage 1 for indicated markers. BLCa82 (**a**) and BLCa85 (**b**) samples.

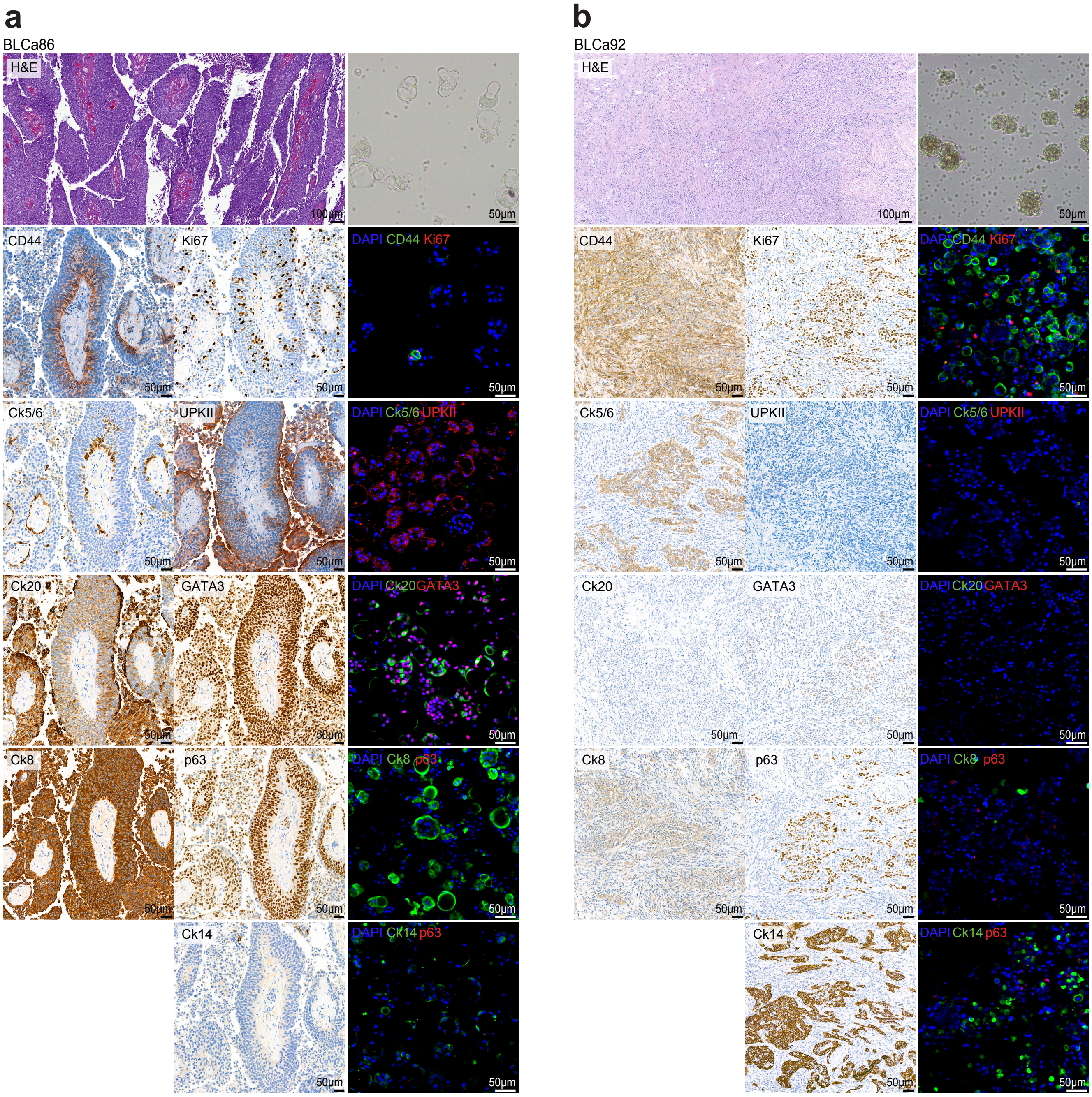

Supplementary Figure 10. Comparison of marker expression between organoids and parental tumor, related to figure 2.

a-b Hematoxylin and Eosin staining and immunohistochemistry staining of parental tumor for indicated markers and brightfield images and whole mount immunofluorescence staining of organoids at passage 1 for indicated markers. BLCa86 (**a**) and BLCa92 (**b**) samples.

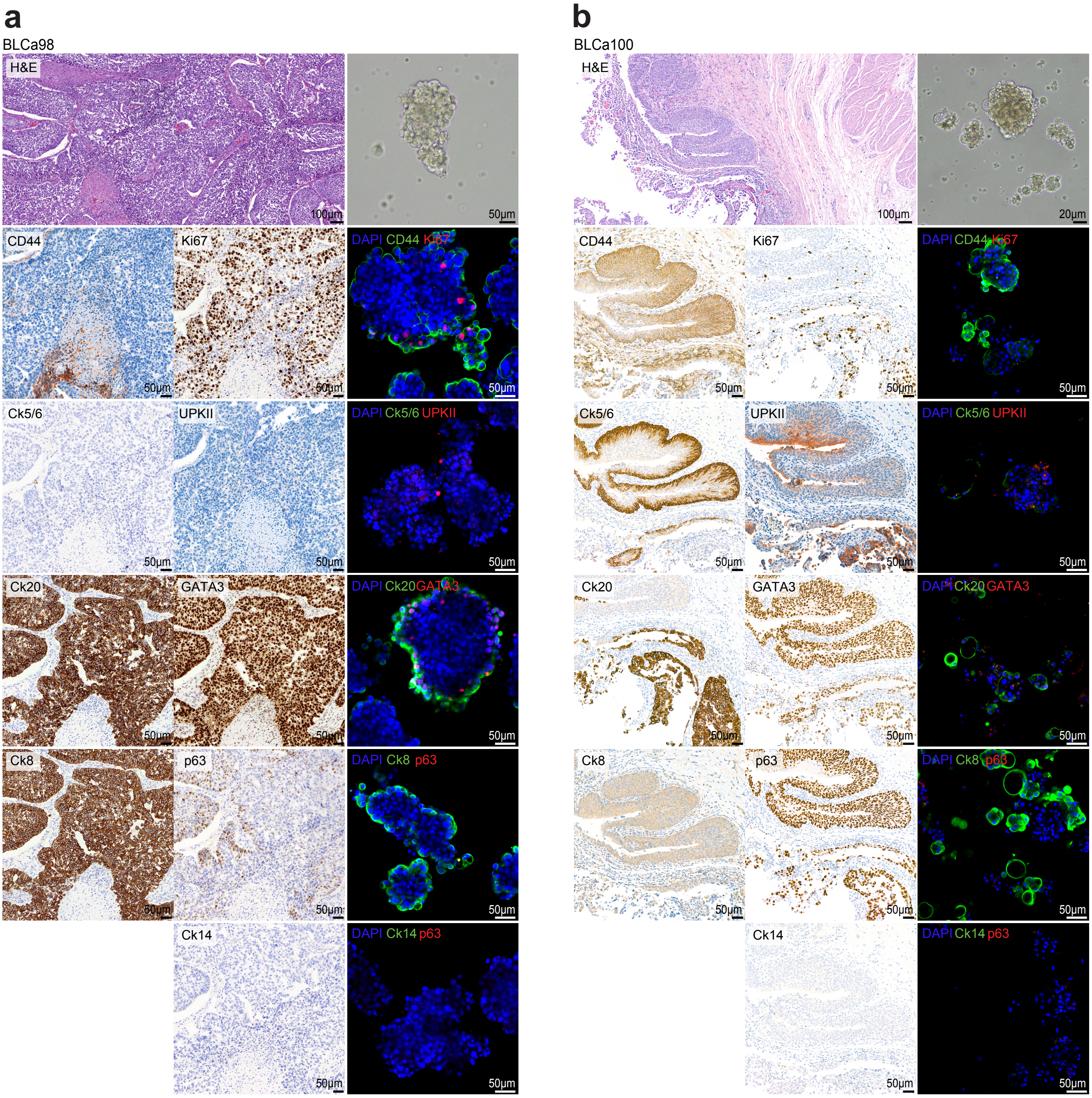

Supplementary Figure 11. Comparison of marker expression between organoids and parental tumor, related to figure 2.

a-b Hematoxylin and Eosin staining and immunohistochemistry staining of parental tumor for indicated markers and brightfield images and whole mount immunofluorescence staining of organoids at passage 1 for indicated markers. BLCa98 (**a**) and BLCa100 (**b**) samples.

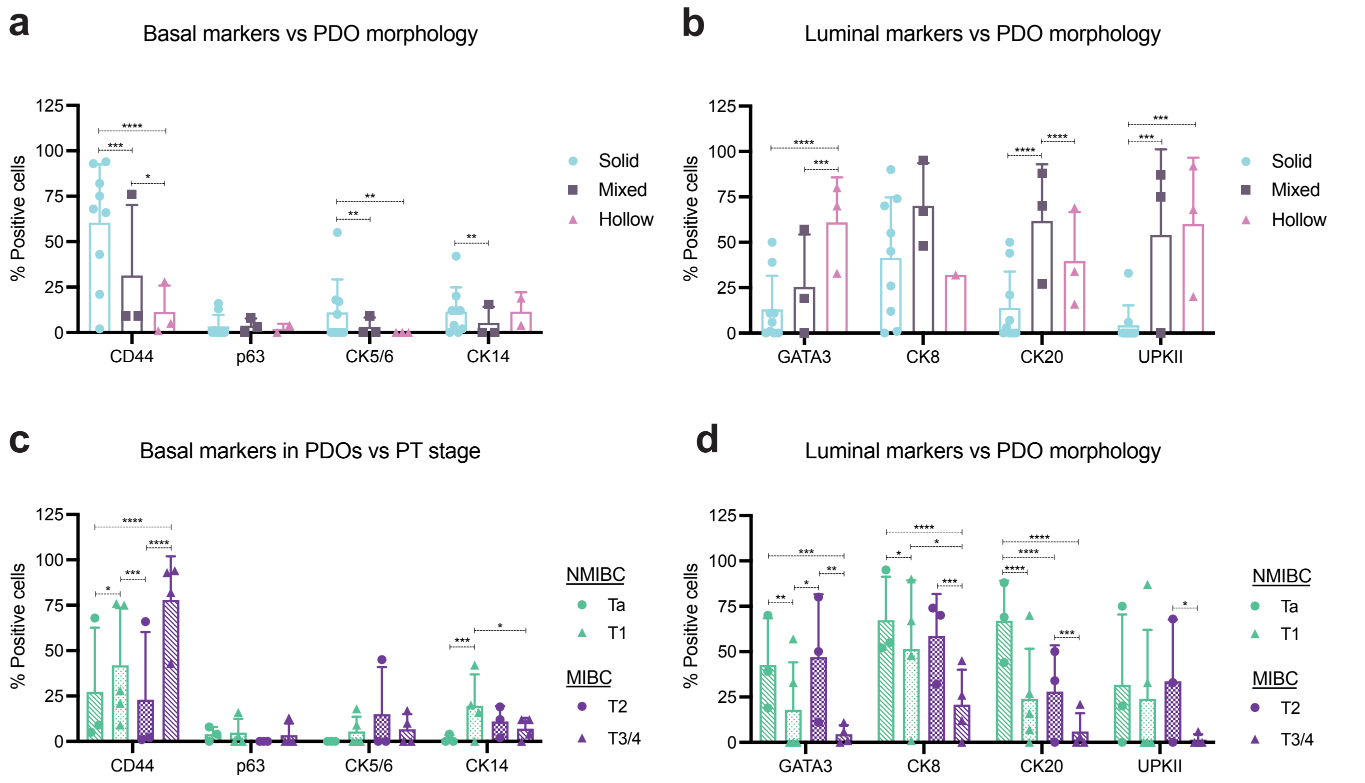

Supplementary Figure 12. Comparison of organoid markers expression with organoid morphology and primary tumor (PT) stage, related to figure 2.

a-d Image quantification of cells positive for basal (**a** and **c**) and luminal (**b** and **d**) markers in patient-derived organoids (PDOs) grouped based on PDO morphology (**a-b**) or PT stage (**c-d**). Each data point corresponds to one biological replicate (i.e., sample, mean biological samples ± SD) calculated from technical replicates (in Source Data) derived from one experiment for each biological sample (n represents the biological sample: n= 9 for solid PDOs, n=3 for mixed PDOs, n=3 for hollow PDOs, n=3 for Ta samples, n=5 for T1 samples, n=3 for T2 tumors, n=4 for T3/4 tumors). Two-way ANOVA test with multi comparison (main column effect) between marker expression and PDO morphologies or PT stages performed between biological replicates, *p-value ≤ 0.05, ** p-value ≤ 0.01, *** p-value ≤ 0.001, **** p-value ≤ 0.0001 (CD44: Solid vs Mixed, p-value = 0.0006; Mixed vs Hollow, p-value = 0.0305; Ta vs T1, p-value = 0.0163; T1 vs T2, p-value = 0.0010. Ck14: Solid vs Mixed, p-value = 0.0054; Ta vs T1, p-value = 0.0010; T1 vs T3/4, p-value = 0.0360. Ck5/6: Solid vs Mixed, p-value = 0.0056; Solid vs Hollow, p-value = 0.0015. GATA3: Mixed vs Hollow, p-value = 0.0003; Ta vs T1, p-value = 0.0022; Ta vs T3/4, p-value = 0.0003; T1 vs T2, p-value = 0.0122; T2 vs T3/4, p-value = 0.0022. Ck20: T2 vs T3/4, p-value = 0.0006. Marker UPKII: Solid vs Mixed, p-value = 0.0003; Solid vs Hollow, p-value = 0.0008; T2 vs T3/4, p-value = 0.0221. Ck8: Ta vs T1, p-value = 0.0122; T1 vs T3/4, p-value = 0.0440; T2 vs T3/4, p-value = 0.0004). Ck, cytokeratin; NMIBC, non-muscle invasive bladder cancer; MIBC, muscle invasive bladder cancer; UPKII, uroplakin II.

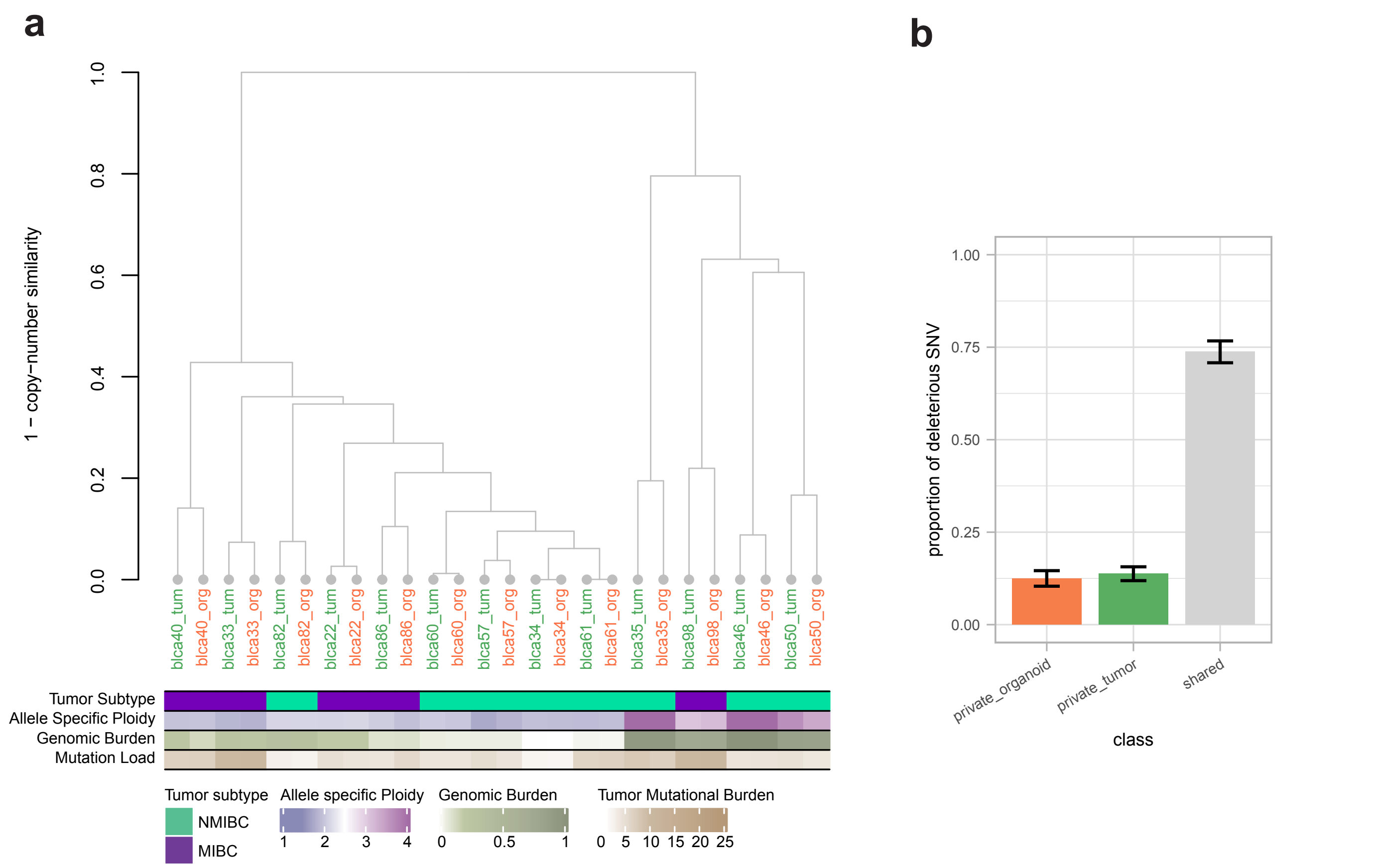

Supplementary Figure 13. Analysis of Whole-Exome Sequencing Data, related to figure 3.

a Graph representing the clustering analysis performed using copy number similarity. Samples information is reported at the bottom of the graph. Two-sided Wilcoxon test, p-value < 2.22e-16. **b** Average proportion of single-nucleotide variants (SNV) across samples reported, stratified per class (shared or private fractions, n=15 biological replicates; mean ± SE). tum, tumor; org, organoids

Supplementary Fig. 14

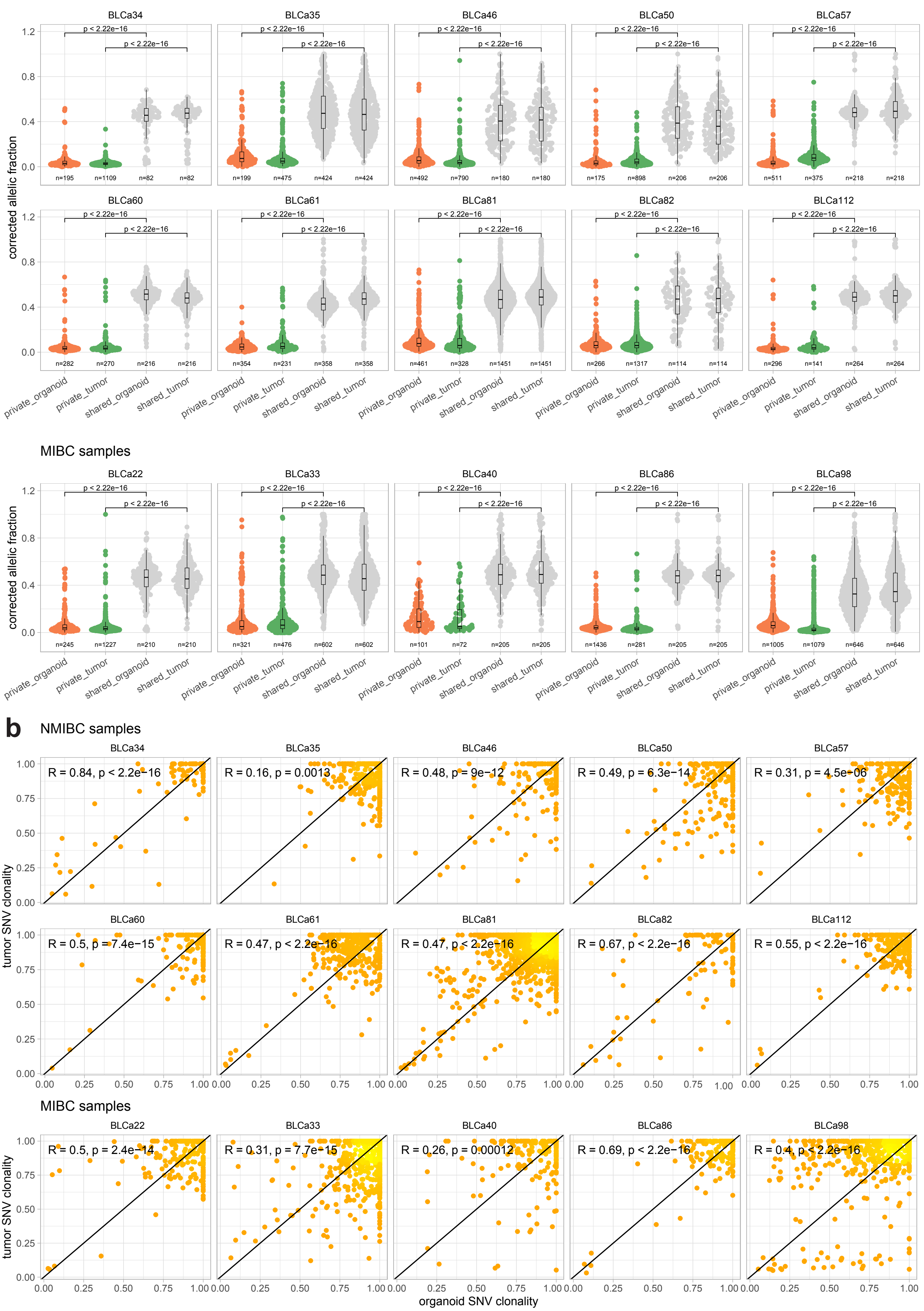

Supplementary Figure 14. Analysis of Whole-Exome Sequencing Data, related to figure 3.

a Graphs representing the purity and ploidy corrected allelic fraction distribution for the shared and private point mutations in organoids and primary tumors. For each sample, the number of SNVs within each class is reported. Box plots indicate median (middle line), 25th, 75th percentile (box) and 5th and 95th percentile (whiskers). Two-sided Wilcoxon-test, * p-value < 2.22e-16. **b** Graphs showing the clonality of sub-clonal point mutations in organoids and primary tumors. Two-sided correlation test p-value and Pearson's correlation coefficient (R) are reported within the figure, p-value < 2.22e-16. NMIBC, non-muscle invasive bladder cancer; MIBC, muscle invasive bladder cancer; SNV, single-nucleotide variant.

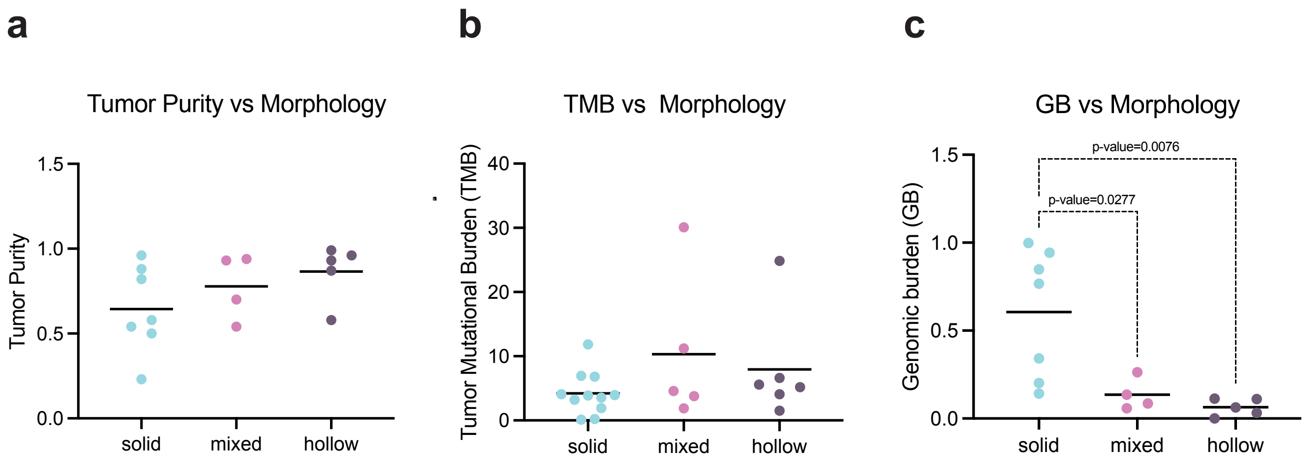

Supplementary Figure 15. Association of genomic features with organoid morphology, related to figure 3.

a-c Analysis of tumor purity (**a**), Tumor Mutational Burden (TMB, **b**), and genomic burden (GB, **c**) in solid, hollow, and mixed organoids. Each point represents one biological replicate (i.e., sample, mean over the samples analysed, n=7 biological samples for solid organoids, n=4 biological samples for mixed organoids (**a, c**), n=5 biological samples for mixed organoids (**b**), and n=5 biological samples for hollow organoids). Ordinary one-way ANOVA with multiple comparison test between the three organoid morpholo-gies.

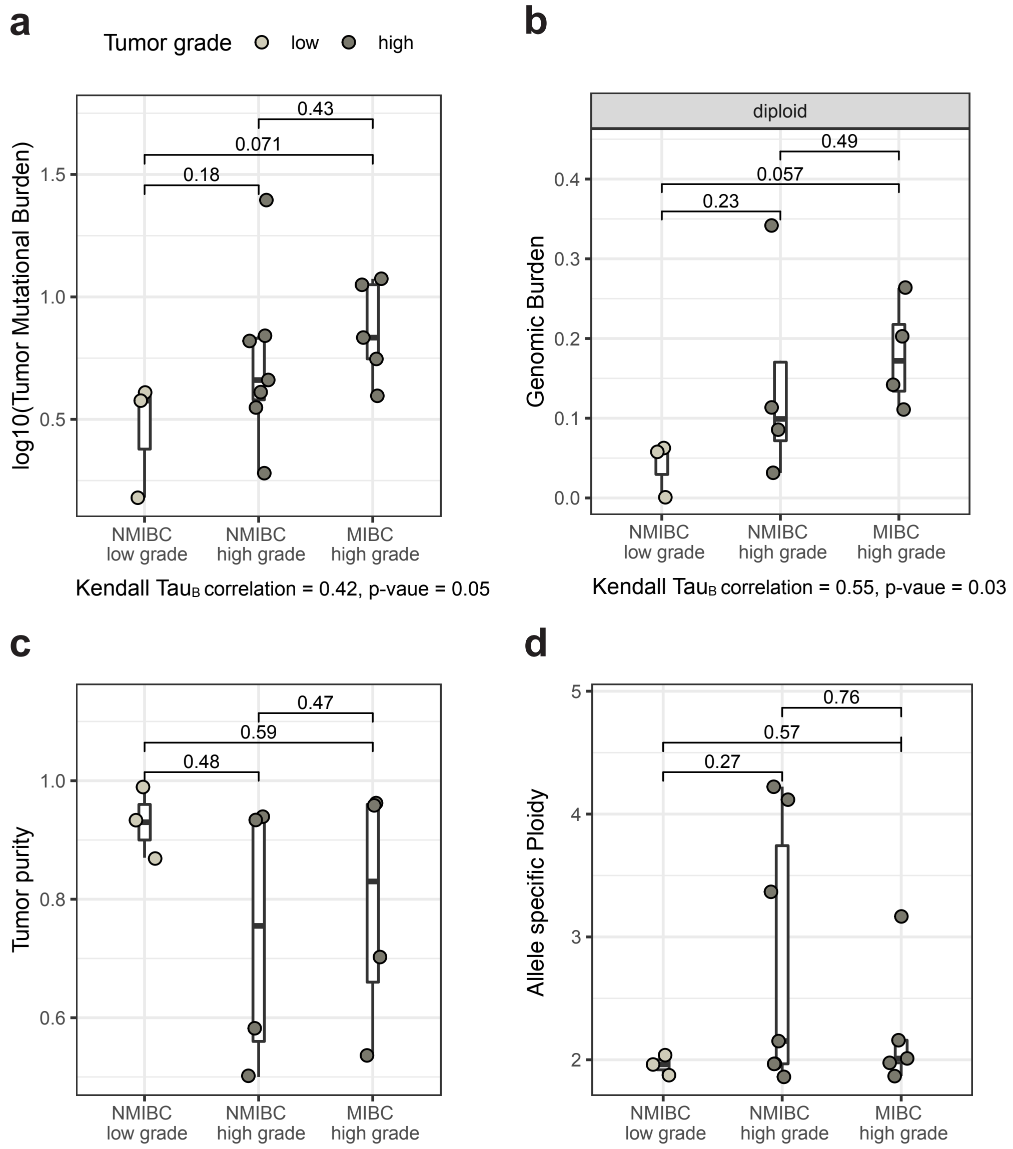

Supplementary Figure 16. Genomic analysis in low and high-grade patient-derived organoids (PDOs), related to figure 4.

a-d Tumor Mutational Burden (**a**, n=15 biological samples), genomic burden (**b**, n=11 biological samples), tumor purity (**c**, n=11 biological samples), and allelic specific ploidy (**d**, n=15 biological samples) in three PDO groups (low-grade non-muscle invasive bladder cancer (NMIBC), high-grade NMIBC, and high-grade muscle invasive bladder cancer (MIBC)). Box plots indicate median (middle line), 25th, 75th percentile (box) and 5th and 95th percentile (whiskers). Two-sided Wilcoxon test was used to test differences between groups and corresponding p-value is reported within the figure. Kendall TauB correlation was computed between the three ordered tumor classes and respective sample-level features (Tumor Mutational Burden and Genomic Burden).

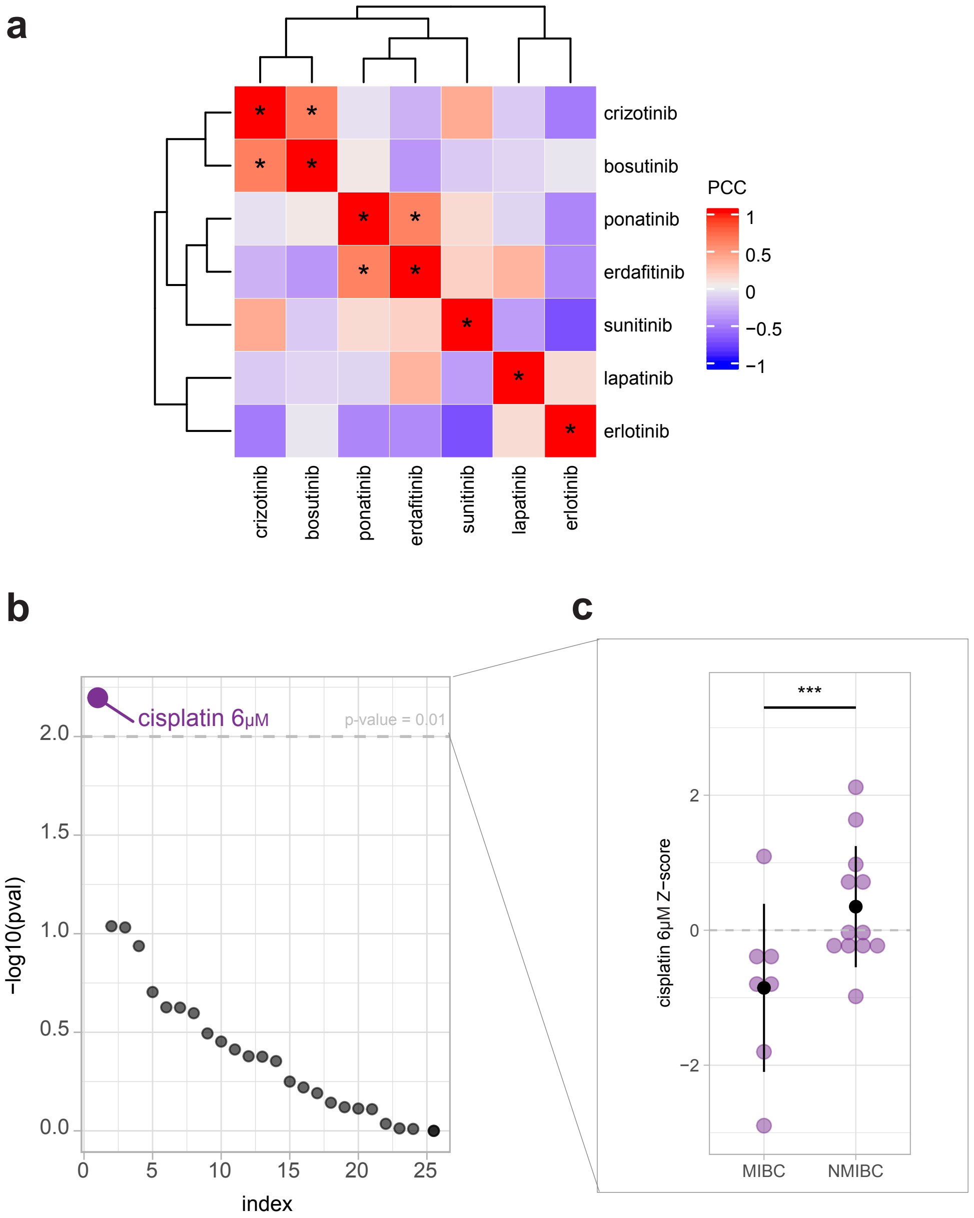

Supplementary Figure 17. Analysis of drug screening data, related to figure 5.

a Correlation matrix showing the Pearson correlation coefficient (PCC) between the different tyrosine-kinases inhibitors tested on organoids. Two-sided correlation test, * p-value ≤ 0.1. **b** Association between drug response and muscle-invasive (MIBC) and non-muscle invasive type (NMIBC). P-value is obtained through Linear Mixed Model (LMM) fit (see Methods). **c** Graphs representing the association between cisplatin 6 µM (left) response and MIBC or NMIBC type. Each data point corresponds to one biological sample (n=7 MIBC samples, n=12 NMIBC samples, mean +/- SD is reported in black) computed as the average z-score among technical replicates. P-value is obtained through LMM fit, ***p-value ≤ 0.001 (see Methods).

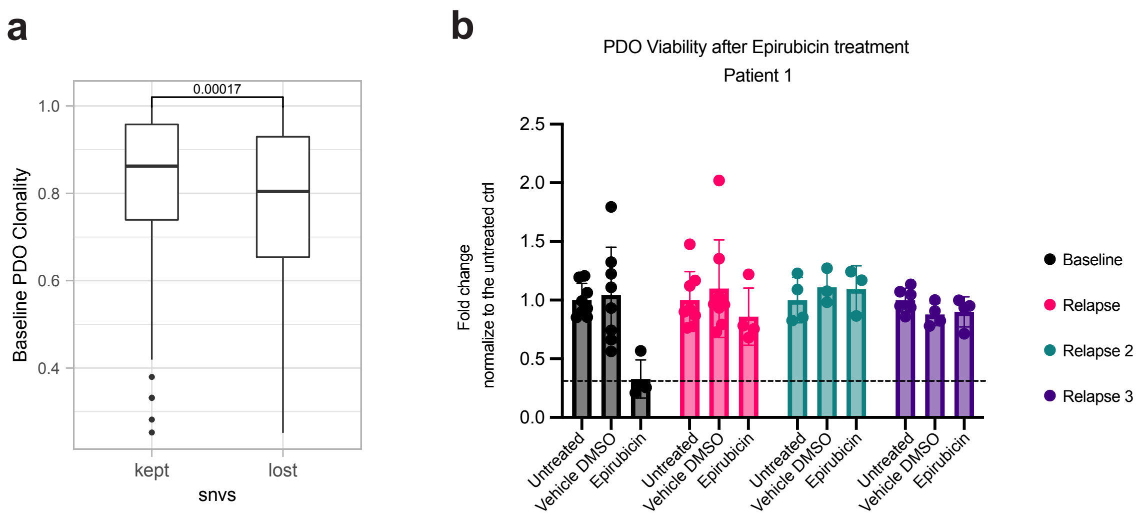

Supplementary Figure 18. Analysis of patient 1, related to figure 6.

a Clonality of single nucleotide variants (SNVs) preserved and lost between organoids from baseline and relapse (n=474 kept SNVs, n=133 lost SNVs). Box plots indicate median (middle line), 25th, 75th percentile (box) and 5th and 95th percentile (whiskers). Two-sided unpaired Wilcoxon test. **b** Organoid longitudinal viability measured in samples derived from patient 1 at different time points (baseline (BLCa69), relapse (BLCa81), relapse 2, and relapse 3 (BLCa136)). The value of organoid viability at 48h after treatment was normalized to the untreated value. Each data point corresponds to one technical replicate (mean± SD) for one experiment (n represents the technical replicates; Baseline and Relapse: n=8 for untreated and vehicle DMSO, and n=4 for epirubicin treatment; Relapse 1: n=4 for untreated, and n=3 for vehicle DMSO and epirubicin treatment; Relapse 2: n=4 for untreated, DMSO vehicle and epirubicin treatment). One-way ANOVA with Dunnet's multiple comparison test between treatment and vehicle DMSO.

Supplementary Information (Supplementary Tables)

Bladder cancer organoids as a functional system to model different disease stages and therapy response

Martina Minoli * and Thomas Cantore*, et al.

	Sample ID	% Ki67 in PT	% Ki67 in PDO
PT > 22% Ki67+ nuclei	BLCa33	35%	ND
	BLCa35	28%	16%
	BLCa40	29%	25%
	BLCa47	26%	0%
	BLCa48	28%	13%
	BLCa50	48%	ND
	BLCa57	35%	ND
	BLCa69	29%	ND
	BLCa81	36%	ND
	BLCa98	43%	7%
PT ≤ 22% Ki67+ nuclei	BLCa22	14%	0%
	BLCa34	20%	1%
	BLCa46	21%	4%
	BLCa60	13%	1%
	BLCa61	18%	ND
	BLCa82	22%	2%
	BLCa85	21%	0%
	BLCa86	18%	4%
	BLCa92	10%	1%
	BLCa100	14%	0%
	BLCa112	6%	6%

Supplementary Table 1. Percentage of Ki67 positive cells in parental tumor (PT) and matched patient-derived organoids (PDO). PT were group based on the median % of $Ki67^+$ cells measured over the total 21 samples (22%). ND, not determined**.**

Supplementary Methods

Bladder cancer organoids as a functional system to model different disease stages and therapy response

Martina Minoli * and Thomas Cantore*, et al.

Supplementary Methods

Single-cell RNA-sequencing

Organoids were collected in Basis medium (100g, 5min) and dissociated into single cells with 1ml TrypLE Express at 37°C for 10 min. Single-cell suspension was counted and cryopreserved in FBS/10%DMSO before single cell RNA sequencing (scRNA-seq). To obtain single cells from tumor tissue, tissues were digested as explained in the protocol for organoid derivation, and then cells were counted and cryopreserved in FBS/10%DMSO before analysis.

Slow-frozen single-cell suspensions were shipped to the Genomics Facility Basel for scRNA-seq processing. Cryovials were thawed and content transferred into 10 mL Basis medium and incubated for 10 min at room temperature to ensure complete removal of DMSO from the cells. Cells were collected (350g, 5 min), resuspended in 1 mL washing buffer consisting of PBS (Ca^{2+}/Mg^{2+}-free, Gibco, 10010-015) and 1% BSA (Sigma-Aldrich, A7906) and sequentially filtered through 100 µm (Falcon, 352360) and 40 µm cell strainers (Falcon, 352340). Apoptotic cells were removed by immunomagnetic cell separation using the Annexin Dead Cell Removal Kit (StemCell Technologies, 17899) and EasySep™ Magnet (StemCell Technologies, 18000). Whenever possible and required, repeat dead cell removal was performed to increase cell viability. Next, cells were washed with a resuspension buffer (PBS with 0.05% BSA), spun down and resuspended in a resuspension buffer. Cell numbers and viability were assessed at each step on a Cellometer K2 Image Cytometer (Nexcelom Bioscience, Cellometer K2) using ViaStain AOPI Staining Solution (Nexcelom Bioscience, CS2-0106-5mL) and PD100 cell counting slides (Nexcelom Bioscience, CHT4-PD100-003). Optimal cell concentrations were set according to 10x Genomics protocols (700-1200 cells/µL). Cells were loaded and processed using the 10x Genomics Chromium platform with the Next GEM Single Cell 3' Reagent Kit v3.1 on 10x Genomics Chromium Single Cell Controller (10x Genomics, PN-120263). 4'000 cells were targeted per sample. Gene expression (GEX) libraries were amplified, pooled and sequenced on the Illumina NovaSeq 6000 platform with 1% PhiX at recommended sequencing depth (20,000-50,000 reads/cell).

Single-cell RNA sequencing analysis

In the following, we briefly describe the overall steps of the sequencing data analysis. Raw reads were mapped to the human reference genome GRCh38 (version 3.0.0 provided by 10X Genomics) using *Cell Ranger*[1] (version 6.0.1). The resulting gene count matrices were used to create a *Seurat*[2] (version 4.1.1) object. Cells with less than one detected feature or more than 50% of mitochondrial reads and features found in less than one cell were removed. Heterotypic doublets were identified and removed with *scDblFinder*[3] (version 1.6.0). Low-quality cells were removed using a median absolute deviation (MAD) threshold of 3. RNA expression was normalized and scaled using *Seurat*. The individual samples were integrated using a reciprocal principal component analysis (rpca). The optimal number of principal components (PCs) was identified with the maxLikGlobalDimEst function from the *intrinsicDimension* (version 1.2.0) R package and a k value of 20. Cell types were annotated using *scMRMA*[4] (version 1.0) and a modified version of the *PanglaoDB*[5] cell marker database. Cell cycle scoring was performed based on the *Seurat* CellCycleScoring function and cell-cycle-related gene sets. Functional enrichment analysis was performed with Enrichr[6] (updated March 29th, 2021) and the Gene Ontology Database[7] (2021 version). The epithelial single cells were classified into molecular subtypes utilizing the consensus molecular classification of MIBC[8] (for samples BLCa98 and BLCa86) and its respective adaptation for NMIBC[9] (for sample BLCa77). The visualizations were generated using the R packages *ggplot2* (version 3.3.6), *ggpubr* (version 0.4.0), *ggrepel* (version 0.9.1), and *pals* (version 1.7).

Organoid characterization, viability, and formation efficiency assays

24 NMIBC and 25 MIBC samples were tested to investigate organoid forming efficiency *in vitro* (Supplementary Data 1). Brightfield images of organoid cultures were analyzed at passage 1 (p1), corresponding to a median culture time of 7 days (3 to 20 days of culture). Organoid morphology was then analyzed in the 40 samples that formed organoids. The total number of organoids per field and their morphology (solid, hollow, or mixed) was manually determined on five brightfield images (technical replicates, Analysis results in Source Data) per sample using cell counter in Fiji[10] (v 2.1.0).

For the viability assay, organoids at p1 were collected and washed in basis medium (100G, 5min) and dissociated into single cells with 1ml TrypLE Express at 37°C for 10 min. Single cells suspension was counted, washed once in basis medium (100g, 5min), and resuspended in BLCa organoid medium. Cells were seeded as replicates in ULA 384 well plate (Corning, cat. No. 4588) in 20 µl of BLCa organoids medium at 8'000-10'000 cells per well. After 48h of culture, 20 µl of fresh BLCa organoids medium was added to each well. After a further 48h, CellTiter-Glo 3D assay (Promega, G9682) was used to determine organoids viability, following manufacturer's instructions. Shortly, 40 µl of CellTiter-Glo 3D reagent were added to each well of the assay. Plates were shaken for 5 min and incubated at 37 °C for 25 min. After incubation, luminescence was measured using Tecan M200 Pro plate reader (Tecan AG).

Drug screening assay

Due to the scarcity of early passage PDOs, only one concentration per drug was tested and was selected based on the maximum plasma concentration for each drug. Most values were extrapolated from phase-I clinical studies, using clinically relevant doses of the tested drug and reported by Liston and Davis[11]. Within SOC, cisplatin was tested at 2 different concentrations as some patients receive a lower dose to prevent excess toxicity. We extrapolated cisplatin concentrations from clinical administration protocols (protocol reg: MPHAURCOIG, https://www.clatterbridgecc.nhs.uk/application/files/8115/9169/7012/Cisplatin__Gemcitabine_Bladder_Cancer-_Full_Dose_Protocol_V1.1.pdf) and converted to a concentration for PDO (cPDO) based on the Cmax from Liston and Davis[11]. Depending on the maximum dose (80 or 70mg/m2), the cPDO is between 6-7 µM. We chose 6 µM to avoid overestimating the PDO response to cisplatin treatment. The choice of using a low *in vitro* concentration of erdafitinib instead was based on the known high drug toxicity and the high affinity to FGFR1,2 and 3 (US Food and Drug Administration. RefID4418085,https://www.accessdata.fda.gov/drugsatfda_docs/nda/2019/212018Orig1s000MultidisciplineR.pdf).

Compound	Company	Catalog No
Bosutinib	Selleckchem	S1014
Cisplatin	Selleckchem	S1166
Crizotinib	Selleckchem	S1068
Daunorubicin	Selleckchem	S3035
Docetaxel	Selleckchem	S1148
Doxorubicin	Selleckchem	S1208
Epirubicin	Selleckchem	S1223
Erlotinib	Selleckchem	S7786
Erdafitinib	Selleckchem	S8401
Everolimus	Selleckchem	S1120
Gemcitabine	Sigma	G6423
Lapatinib	Selleckchem	S2111
Methotrexate	Selleckchem	S1210
Mitomycin C	Selleckchem	S8146
Olaparib	Selleckchem	S1060
Paclitaxel	Selleckchem	S1150
Ponatinib	Selleckchem	S1490
Rapamycin	Selleckchem	S1039
Sunitinib	Selleckchem	S7781
Temsirolimus	Selleckchem	S1044
Vinblastine	Selleckchem	S4505

Supplementary Table 2. Drugs used in organoid drug screening.

Drugs association analyses

Drug screening z-scores were used to perform association analyses with both PDOs genomic and phenotypic information. For the former, for every drug and for every corresponding target gene (Analysis results in Source Data), we tested the association between drugs activity and gene mutational status by fitting a random intercept Linear Mixed Model (LMM): $z \sim \beta_1 x + \beta_0 m + \epsilon$, where *z* is a vector of z-scores for a drug *d* across tested PDOs replicates from different samples, *x* is a binary vector encoding the mutation status of each PDO (0 if *wild-type* and 1 if *mutated*) and *m* is a vector encoding the PDO sample to which each replicate belongs, treated as a random effect. Estimated β_1 was considered as the effect-size of the resulting association. Corresponding p-values were obtained from a Likelihood Ratio Test comparing the shown model, to a null one (lacking of $\beta_1 x$ term).

Three classes of genomic aberrations were considered: copy-gain (one of gain, gain_unb, gain_del, amp, amp_unb, amp_del), copy-loss (one of homo_del, hemi_del) and deleterious SNVs. Only drug-target pairs with at least 3 aberrant and 3 wild-type models were tested. The same strategy was adopted for the drug-phenotype association analysis, where the binary vector x encoded the phenotype information of two compared groups (*solid vs. hallow morphology*, *MIBC* vs *NMIBC*, Analysis results in Source Data). PDO morphology was defined by the most prevalent morphology observed in the organoid cultures. SNVs-Enriched-pathway association analysis (Fig. 5c) was performed by considering, for each frequently reactome term previously identified (see Deleterious SNVs and SNVs enrichment analysis), the presence or absence of enrichment, for each PDO. FDR

correction was then performed. The binary matrix reported in Supplementary Data 5 reports the groups considered for each term.

Parental tumor histology

FFPE tissue from the tumor samples was sectioned and stained by hematoxylin, eosin (H&E), and immunohistochemistry (IHC, Supplementary Table 3). Brightfield images of tissue sections were acquired with slide scanner (3DHistech Pannoramic 250 Flash II). Experiments were performed by the Translational Research Unit (TRU) at the Institute of Pathology, University of Bern or performed in house for marker CD44 and UPKII. For IHC, antigen retrieval was performed in citrate buffer for 10 min, followed by blocking of endogenous peroxidases in 30% H_2O_2 for 10min. Primary antibody UPKII and CD44 were diluted 1to500 in 1% BSA/PBS/0.05% Tween. Staining of rabbit primary antibodies was developed using the anti-rabbit EnVision+System-HRP (DAKO, K4003) and staining of mouse primary antibodies was developed using the anti-mouse EnVision+System-HRP (DAKO, K4001) following manufacturer's instructions.

Image analysis was processed in QuPath v.0.2.3 (Queen's University, Belfast, Northern Ireland). The percentage of positive cells was calculated based on the detected nuclei. PTs were grouped in ≤ or >22% of $Ki67^+$ nuclei per section (low- and high-proliferation rate, respectively). A cut-off of 22% of $Ki67^+$ nuclei per section was defined as the median value between all the samples analyzed (Supplementary Table 1).

	Company	Catalog No	Clone
Mouse monoclonal Anti-CD44	BD Pharmingen	550988	515
Mouse monoclonal Anti-Ck5/6	Merck & Cie	MAB1620	D5/16
Mouse monoclonal Anti-Ck8	BD Bioscience	345779	CY-90
Mouse monoclonal Anti-Ck14	Biosystems	NCL-L-LL002	LL002
Mouse monoclonal Anti-CK20	Biosystems	320M-16	Ks20.8
Mouse monoclonal Anti-GATA3	Biosystems	390M-14	L50-823
Rabbit monoclonal Anti-Ki67	Biosystems	RM-9106-S1	SP6
Mouse monoclonal Anti-p63	Biosystems	NCL-L-p63	7JUL
Rabbit monoclonal Anti-UPKII	Abcam	ab213655	EPR18799

Supplementary Table 3. Antibodies for immunohistochemistry. Ck, cytokeratin; UPKII, uroplakin II.

Whole-mount immunofluorescence staining of organoids

Organoids were collected in Basis medium and spin down (100g, 5min). Organoids were suspended in 1xPBS and collected in a 96Well Round (U) Bottom plate (Sigma, 92097) and spin down (100g, 5min). Medium was removed and organoids fixed in 4% paraformaldehyde (PFA) for 30min at room temperature. PFA was removed and organoids washed three times in 1xPBS (100g, 5min). Organoids were then permeabilized in 0.3% Triton-X for 10min at room temperature and then blocked in 10% donkey serum in PBS/0.05% Tween for 1h at room temperature gently rocking. After the blocking, the organoids were incubated with primary antibodies (1to100, Supplementary Table 4) in blocking solution overnight at 4°C. After incubation with primary antibodies, organoids were washed three times (100g, 5min) in PBS/0.05% Tween. Organoids were then incubated with secondary antibodies AlexaFluor 555 donkey anti-rabbit (1to200), AlexaFluor 488 donkey anti-mouse (1to200) and DAPI (1to1000) in blocking solution for 2h at room temperature gently rocking. At the end, organoids were washed three times (100g, 5min) in PBS/0.05% Tween-20, gently rocking and transferred to flat glass

bottom, black walled 96-well plates (Corning, cat. No. 4580) in PBS/0.05% Tween-20. Immunofluorescence staining was imaged using a confocal microscope (ZEISS LSM 710 with Airyscan). Image analysis was performed with QuPath v.0.2.3 (Queen's University, Belfast, Northern Ireland) and Fiji[10] (v 2.1.0). The percentage of positive cells was calculated based on the marker of reference DAPI (Analysis results in Source Data).

	Company	Catalog No	Clone
Mouse monoclonal Anti-CD44	BD Pharmingen	550988	515
Rabbit polyclonal Anti-Ck5/6	BioLegend	905501	Poly 19055
Mouse monoclonal Anti-Ck8	Thermo Fisher Scientific	MA1-06318	M20
Mouse monoclonal Anti-Ck14	Abcam	ab9220	RCK107
Mouse monoclonal Anti-Ck20	Abcam	Ab854	Ks20.8
Rabbit monoclonal Anti-GATA3	Cell Signalling	5852	D13C9
Rabbit monoclonal Anti-Ki67	Gene Tex	GTX16667	SP6
Rabbit monoclonal Anti-p63	Abcam	ab124762-100	EPR5701
Rabbit monoclonal Anti-UPKII	Abcam	ab213655	EPR18799
Donkey anti-mouse IgG, Alexa Fluor 488	Thermo Fisher Scientific	A21202	-
Donkey anti-rabbit IgG, Alexa Fluor 555	Thermo Fisher Scientific	A21434	-
DAPI	Thermo Fisher Scientific	62248	-

Supplementary Table 4. Antibodies for immunofluorescence. Ck, cytokeratin; UPKII, uroplakin II.

Statistics and Reproducibility

Unless specified, all statistical analysis was performed using GraphPad Prism (v 9.2.0) and R (V.4.0.3, R Core Team, 2016). Statistical tests applied throughout the study that are not explained in detail in this section, are reported in results, figure legends, and in the methods accordingly.

In Fig. 1e, an unpaired two-sided Wilcoxon test was used to test cell viability difference between NMIBC and MIBC PDO (an average value of cell viability for each sample was used as replicate, n = 5 biological replicates for both NMIBC and MIBC). In Fig. 2c, two-way ANOVA with Tukey's multiple comparison test (matching values of each biological sample stacked into sub-columns) was used to compare the % of PDO morphologies between tumor stages and grades. An average value was calculated for each biological replicate from the evaluation of an average of 5 figures for each sample. The reported micrographs in supplementary Fig.1 and 2 are representative images of one experiment and are representative image of the results reported in Fig.2c.

In Fig. 5a, 6e and 6j, one-way ANOVA test was used to compare each drug treatment to its vehicle (p-value was adjusted within samples controlled for vehicle, i.e., H_2O or DMSO). Effective compounds were selected based on a z-score ≤ to -1.5 and statistically lower (adjusted p-value < 0.05) than the vehicle condition. Z-score for each sample was calculated from n=average of 7 replicates for untreated and vehicles, and n=average of 3 replicates for each drug depending on the biological material available. To test the effect of one drug in two different samples, z-scores were compared with a nonparametric two-sided Wilcoxon test.

In Supplementary Fig. 12, two-way ANOVA test with Tukey's multiple comparison was used to compare the percentage of positive cells between three groups (solid, hollow, or mixed morphologies) or four groups (Ta, T1, T2, and T3 tumor-stages). For statistical analysis, the average value for the positive cells, the standard deviation calculated from the evaluation of technical replicates, and the number of replicates were used for each biological sample.

The micrographs showing immunofluorescence, immunohistochemistry staining and or brightfield images of organoids and parental tumor showed in Fig. 1c-d, 2a, 2d-e, and supplementary Fig. 3a-b, 4, and 5-11 are representative of one experiment performed for each biological sample.

References

1. Zheng, G. X. Y. *et al.* Massively parallel digital transcriptional profiling of single cells. *Nat Commun* **8**, 14049 (2017).
2. Hao, Y. *et al.* Integrated analysis of multimodal single-cell data. *Cell* **184**, 3573-3587.e29 (2021).
3. Germain, P.-L., Lun, A., Meixide, C. G., Macnair, W. & Robinson, M. D. Doublet identification in single-cell sequencing data using *scDblFinder*. Preprint at https://doi.org/10.12688/f1000research.73600.2 (2022).
4. Li, J., Sheng, Q., Shyr, Y. & Liu, Q. scMRMA: single cell multiresolution marker-based annotation. *Nucleic Acids Research* **50**, e7 (2022).
5. Franzén, O., Gan, L.-M. & Björkegren, J. L. M. PanglaoDB: a web server for exploration of mouse and human single-cell RNA sequencing data. *Database* **2019**, baz046 (2019).
6. Kuleshov, M. V. *et al.* Enrichr: a comprehensive gene set enrichment analysis web server 2016 update. *Nucleic Acids Research* **44**, W90–W97 (2016).
7. The Gene Ontology Consortium. The Gene Ontology resource: enriching a GOld mine. *Nucleic Acids Research* **49**, D325–D334 (2021).
8. Kamoun, A. *et al.* A Consensus Molecular Classification of Muscle-invasive Bladder Cancer. *European Urology* (2019) doi:10.1016/j.eururo.2019.09.006.
9. Lindskrog, S. V. *et al.* An integrated multi-omics analysis identifies prognostic molecular subtypes of non-muscle-invasive bladder cancer. *Nat Commun* **12**, 2301 (2021).
10. Schindelin, J. *et al.* Fiji: an open-source platform for biological-image analysis. *Nat Methods* **9**, 676–682 (2012).
11. Liston, D. R. & Davis, M. Clinically relevant concentrations of anticancer drugs: A guide for nonclinical studies. *Clin Cancer Res* **23**, 3489–3498 (2017).
