# Supplementary material for: Bladder cancer organoids as a functional system to model different disease stages and therapy response
Source: Nat Commun. 2023 Apr 18;14:2214. doi: 10.1038/s41467-023-37696-2 (PMC10113240; doi:10.1038/s41467-023-37696-2)
Supplement: Supplementary file 2 — Reporting Summary [file 41467_2023_37696_MOESM2_ESM.pdf]

nature portfolio

Corresponding author(s): Marianna Kruithof-de Julio
Last updated by author(s): 06.02.2023

Reporting Summary

Nature Portfolio wishes to improve the reproducibility of the work that we publish. This form provides structure for consistency and transparency in reporting. For further information on Nature Portfolio policies, see our Editorial Policies and the Editorial Policy Checklist.

Statistics

For all statistical analyses, confirm that the following items are present in the figure legend, table legend, main text, or Methods section.

n/a	Confirmed	
☐	☒	The exact sample size (*n*) for each experimental group/condition, given as a discrete number and unit of measurement
☐	☒	A statement on whether measurements were taken from distinct samples or whether the same sample was measured repeatedly
☐	☒	The statistical test(s) used AND whether they are one- or two-sided<br>*Only common tests should be described solely by name; describe more complex techniques in the Methods section.*
☐	☒	A description of all covariates tested
☐	☒	A description of any assumptions or corrections, such as tests of normality and adjustment for multiple comparisons
☐	☒	A full description of the statistical parameters including central tendency (e.g. means) or other basic estimates (e.g. regression coefficient) AND variation (e.g. standard deviation) or associated estimates of uncertainty (e.g. confidence intervals)
☐	☒	For null hypothesis testing, the test statistic (e.g. *F*, *t*, *r*) with confidence intervals, effect sizes, degrees of freedom and *P* value noted<br>*Give P values as exact values whenever suitable.*
☒	☐	For Bayesian analysis, information on the choice of priors and Markov chain Monte Carlo settings
☐	☒	For hierarchical and complex designs, identification of the appropriate level for tests and full reporting of outcomes
☐	☒	Estimates of effect sizes (e.g. Cohen's *d*, Pearson's *r*), indicating how they were calculated

Our web collection on statistics for biologists contains articles on many of the points above.

Software and code

Policy information about availability of computer code

Data collection

Cell viability data were collected using Tecan M200 Pro plate reader (i-control™). Single cell RNA sequencing data were obtained with the NovaSeq platform. Whole exome sequencing were obtained with Illumina NovaSeq 6000.

Data analysis

All software used was either open source or commercially available.

Statistical analyses were performed using GraphPad Prism (v 9.2.0) and R (v 4.0.3). Brightfield images of tissue sections were acquired with slide scanner (3DHistech Pannoramic 250 Flash II). Immunofluorescence staining was imaged using a confocal microscope (ZEISS LSM 710 with Airyscan). Image analysis was performed with QuPath (v.0.2.3 Queen's University, Belfast, Northern Ireland) and Fiji (v 2.1.0103).

Single cells RNA analysis In the following, we briefly describe the overall steps of the sequencing data analysis. Raw reads were mapped to the human reference genome GRCh38 (version 3.0.0 provided by 10X Genomics) using Cell Ranger (version 6.0.1). The resulting gene count matrices were used to create a Seurat (version 4.1.1) object. Cells with less than one detected feature or more than 50% of mitochondrial reads and features found in less than one cell were removed. Heterotypic doublets were identified and removed with scDblFinder (version 1.6.0). Low-quality cells were removed using a median absolute deviation (MAD) threshold of 3. RNA expression was normalized and scaled using Seurat. The individual samples were integrated using a reciprocal principal component analysis (rpca). The optimal number of principal components (PCs) was identified with the maxLikGlobalDimEst function from the intrinsicDimension (version 1.2.0) R package and a k value of 20. Cell types were annotated using scMRMA (version 1.0) and a modified version of the PanglaoDB cell marker database. Cell cycle scoring was performed based on the Seurat CellCycleScoring function and cell-cycle-related gene sets. Functional enrichment analysis was performed with Enrichr (update March 29th 2021) and the Gene Ontology Database (2021 version). The epithelial single cells were classified into molecular subtypes utilizing the consensus molecular classification of MIBC8 (for samples BLCa98 and BLCa86, Consensus classifier) and its respective adaptation for NMIBC9 (for sample BLCa77, UROMAL2021 classifier). The visualizations were generated using the R packages

ggplot2 (version 3.3.6), ggpubr (version 0.4.0), ggrepel (version 0.9.1), and pals (version 1.7).

Whole exome sequencing analysis: FASTA files were trimmed using Trimmomatic, quality checked performed with FastQC, and reads were aligned with BWA algorithm on hg38. Deduplication, realignment around indels and base recalibration were then performed using GATK4. Mutation, copy-number data and samples level statistics were obtained through the recently established SPICE analysis pipeline. Briefly, it includes quality control step to assess the similarity between matched samples by running SPIA, allele-specific copy number assessment upon data segmentation by running CLONET v2 and mutation and annotation calling via MuTect2 and VEP

Code generated to visualize the whole exome sequencing data are readily available upon request.

For manuscripts utilizing custom algorithms or software that are central to the research but not yet described in published literature, software must be made available to editors and reviewers. We strongly encourage code deposition in a community repository (e.g. GitHub). See the Nature Portfolio guidelines for submitting code & software for further information.

Data

Policy information about availability of data

All manuscripts must include a data availability statement. This statement should provide the following information, where applicable:

- Accession codes, unique identifiers, or web links for publicly available datasets
- A description of any restrictions on data availability
- For clinical datasets or third party data, please ensure that the statement adheres to our policy

All unique materials are readily available upon request to the corresponding author.

Databases/datasets generated:
- Whole exome sequencing data generated in this study have been deposited in dbGAP and are available under the accession number phs003149.v1.p1.
- Allele specific copy-number and SNVs calls derived from Whole Exam Sequencing are available on GitHub (https://github.com/demichelislab/BLCa_organoids_data).
-Single cells RNAseq was deposited in Gene Expression Omnibus (GEO) and is available under the accession number GSE217956.

Databases/datasets used:
-human reference genome GRCh38 (version 3.0.0 provided by 10X Genomics) using Cell Ranger (version 6.0.1)
-Allele-specific TCGA data of MIBC BLCA patients used in this paper were obtained from (https://zenodo.org/record/5266542#.Y97dDi-B1sE) (Ciani et al 2022)
-MSK genomics data of NMIBC patients were downloaded from cBioPortal (https://www.cbioportal.org).

Human research participants

Policy information about studies involving human research participants and Sex and Gender in Research.

Reporting on sex and gender	Clinical details of the patients included in this study are reported in Table 2 and supplementary Table 1. The patient cohort comprised 34 males and 4 females. Sex- and gender-based analyses were not performed in this study because out of scope.
Population characteristics	Clinical details of the patients included in this study are reported in Table 2. At the time of the sampling patients were at 42 to 91 years of age (median of 69 years). 34 males and 4 females.
Recruitment	Participants who underwent TUR-B, cystectomy or nephroureterectomy at the Inselspital, University Hospital in Bern were recruited in this study after providing written informed consent. Selection was bias was limited as much as possible, however the study population was recriited on a single-center bias, enriching for a population of caucasian men
Ethics oversight	All analyses were carried out in accordance with protocol approved by the Ethical Committee Bern (Cantonal Ethical approval KEK 06/03 and 2017-02295)

Note that full information on the approval of the study protocol must also be provided in the manuscript.

Field-specific reporting

Please select the one below that is the best fit for your research. If you are not sure, read the appropriate sections before making your selection.

☒ Life sciences ☐ Behavioural & social sciences ☐ Ecological, evolutionary & environmental sciences

For a reference copy of the document with all sections, see nature.com/documents/nr-reporting-summary-flat.pdf

Life sciences study design

All studies must disclose on these points even when the disclosure is negative.

Sample size	We included 49 samples from 38 patients. From a subset of samples (n=22) that passed molecular QC, we performed a deeper characterization (e.g., WES, drug screening, proliferation). The sample size was determined basing on the recruitment capacity of the study as well as on biological material availability. Multiple independent measurements were taken for each patient-derived sample (drug screening,

genetic sequencing, organoid formation assay). Sample size for the performed assays was optimized based on expected mean differences and according to assay manufacturer's indications (where applicable) and on previous experiments performed with organoids derived from patient material (Karkampouna et al. 2021, 10.1038/s41467-021-21300-6).

Data exclusions	From cell viability data, outliers data were identified with GraphPad Prism (v 9.2.0, ROUT method, FDR or Q = 1%) and excluded.
Replication	Each in vitro experiment, including RNA and WES, was performed as n=1, considering each patient an independent biological replicate, all attempts to replicate in vitro experiments were successful. Technical replicates were used for drug screening assay and viability assay and were selected based on biological material availability. For each drug screening assays an average of 7 technical replicates for untreated and vehicles, and an average of 3 replicates for each drug condition were seeded (based on biological material availability). For each viability assay instead replicates were selected based on biological availability (BLCa30 n = 10, for BLCa34, BLCa51, BLCa22, BLCa26 and BLCa53 n = 8, for BLCa60 and BLCa86 n = 4, for BLCa40 n = 7, for BLCa77 n = 6).
Randomization	For the in vitro organoid drug screening, blanks and functional controls were consistently included to reduce the effect of confounders and covariates. Randomization was not relevant for this study, which was a direct comparison of organoids derived from bladder cancer and primary tumor.
Blinding	Bliding was not relevant for this study, which was a direct comparison of organoids derived from bladder cancer and primary tumor. For molecular analyses, data collection and analysis were performed by different investigators to minimize bias.

Reporting for specific materials, systems and methods

We require information from authors about some types of materials, experimental systems and methods used in many studies. Here, indicate whether each material, system or method listed is relevant to your study. If you are not sure if a list item applies to your research, read the appropriate section before selecting a response.

Materials & experimental systems

n/a	Involved in the study
☐	☒ Antibodies
☒	☐ Eukaryotic cell lines
☒	☐ Palaeontology and archaeology
☒	☐ Animals and other organisms
☒	☐ Clinical data
☒	☐ Dual use research of concern

Methods

n/a	Involved in the study
☒	☐ ChIP-seq
☒	☐ Flow cytometry
☒	☐ MRI-based neuroimaging

Antibodies

Antibodies used	Antibodies for immunofluroescence: Mouse monoclonal Anti-CD44 BD Pharmingen 550988 (clone 515), Rabbit polyclonal Anti-CK5/6 BioLegend 905501 (clone Poly 19055), Mouse monoclonal Anti-CK8 Thermo Fisher Scientific MA1-06318 (clone M20), Mouse monoclonal Anti-CK14 Abcam ab9220 (clone RCK107), Mouse monoclonal Anti-CK20 Abcam Ab854 (clone Ks20.8), Rabbit monoclonal Anti-GATA3 Cell Signalling 5852 (clone D13C9), Rabbit monoclonal Anti-Ki67 Gene Tex GTX16667 (clone SP6), Rabbit monoclonal Anti-p63 Abcam ab124762-100 (clone EPR5701), Rabbit monoclonal Anti-UPKII Abcam ab213655 (clone EPR18799), Donkey anti-mouse IgG, Alexa Fluor 488 Thermo Fisher Scientific A21202, Donkey anti-rabbit IgG, Alexa Fluor 555 Thermo Fisher Scientific A21434, DAPI Thermo Fisher Scientific 62248.<br>Antibodies for immunohistochemistry: Mouse monoclonal Anti-CD44 BD Pharmingen 550988 (clone 515), Mouse monoclonal Anti-CK5/6 Merck& Cie MAB1620 (clone D5/16 B4), Mouse monoclonal Anti-CK8 BD Bioscience 345779 (clone CY-90), Mouse monoclonal Anti-CK14 Biosystems NCL-L-LL002 (clone LL002), Mouse monoclonal Anti-CK20 Biosystems 320M-16 (clone Ks 20.8), Mouse monoclonal Anti-GATA3 Biosystems 390M-14 (clone L50-823), Rabbit monoclonal Anti-Ki67 Biosystems RM-9106-S1 (clone SP6), Mouse monoclonal Anti-p63 Biosystems NCL-L-p63 (clone 7JUL), Rabbit monoclonal Anti-UPKII Abcam ab213655 (clone EPR18799)
Validation	The concentration and method specifications (e.g. antigen retrieval) were used based on the protocols of the antibody manufacturer`s. Immunohistochemistry stainings were performed by the Translational Research Unit (TRU) at the Institute of Pathology, University of Bern where all antibodies were previously validated for human diagnostic purposes. For IF applications, isotype controls matching the primary antibodies were used as negative controls.<br>Ki67: validated on human cervical carcinoma and by Murai et al., PMID: 26965827<br>CD44: human specific, validate by Kansas et al., PMID: 1702327<br>p63: validated on prostate tissue, https://doi.org/10.1073/pnas.0510652103<br>Ck5/6: human specific, validated on breast cancer tissue by Kittrell et al. 2011, PMID:21466693<br>CK14: validated on bladder cancer tissue by Li et al. 2019, PMID: 29350066<br>Ck20: human specific, validated on intestinal epithelium by Wang et al. 2017, PMID: 27935584<br>GATA3: validated on trophoblast cells by Okae et al. 2018, https://doi.org/10.1016/j.stem.2017.11.004<br>UPKII: human specific, validated on human urothelial cells by Rashidbenam et al. 2021, PMID: 33805910<br>CK8: validated by knockdown as per reports available at the manufacturer's website. Positive control used: frozen sections human colon
