# Supplementary material for: Bladder cancer organoids as a functional system to model different disease stages and therapy response
Source: Nat Commun. 2023 Apr 18;14:2214. doi: 10.1038/s41467-023-37696-2 (PMC10113240; doi:10.1038/s41467-023-37696-2)
Supplement: Supplementary file 3 — Description supplementary files [file 41467_2023_37696_MOESM3_ESM.pdf]

Description of Additional Supplementary Files

Supplementary Data 1
Description: List of bladder cancer samples used to test organoids derivation. Information regarding age (at sampling), sex, specimen type, tumor stage and grade, and organoid formation are reported.

Supplementary Data 2
Description: Genomic measures and statistics computed on samples (tumor and/or matched PDOs) sequenced with WES.

Supplementary Data 3
Description: Comparison of frequency of mutations (snv, homo-deletion, hemi-deletion, gain, amp) for a list of genes between our non-muscle invasive cohort and the Memorial Sloan Kettering (MSK) cohort.

Supplementary Data 4
Description: Comparison of frequency of mutations (snv, homo-deletion, hemi-deletion, gain, amp) for a list of genes between our muscle invasive cohort and the The Cancer Genome Atlas (TCGA) cohort.

Supplementary Data 5
Description: Enrichment analysis on deleterious single nucleotide variants (SNVs) of 20 bladder cancer PDOs. 0=not significantly enriched, 1=significantly enriched.

Supplementary Data 6
Description: Replicate-level drug screening data for 21 bladder cancer organoids.

Supplementary Data 7
Description: Sequencing statistics of 22 bladder cancer samples (tumor and/or matched PDOs).
